# DIALing-up the preclinical characterization of gene-modified adoptive cellular immunotherapies

**DOI:** 10.3389/fimmu.2023.1264882

**Published:** 2023-11-28

**Authors:** Maria Letizia Giardino Torchia, Gordon Moody

**Affiliations:** ^1^ Cell Therapy Unit, Oncology Research, AstraZeneca, Cambridge, United Kingdom; ^2^ Cell Therapy Unit, Oncology Research, AstraZeneca, Gaithersburg, MD, United States

**Keywords:** CAR-T cells, immunotherapy, adoptive cell therapy, tumor microenvironment, animal models

## Abstract

The preclinical characterization of gene modified adoptive cellular immunotherapy candidates for clinical development often requires the use of mouse models. Gene-modified lymphocytes (GML) incorporating chimeric antigen receptors (CAR) and T-cell receptors (TCR) into immune effector cells require *in vivo* characterization of biological activity, mechanism of action, and preclinical safety. Typically, this characterization involves the assessment of dose-dependent, on-target, on-tumor activity in severely immunocompromised mice. While suitable for the purpose of evaluating T cell-expressed transgene function in a living host, this approach falls short in translating cellular therapy efficacy, safety, and persistence from preclinical models to humans. To comprehensively characterize cell therapy products in mice, we have developed a framework called “DIAL”. This framework aims to enable an end-to-end understanding of genetically engineered cellular immunotherapies *in vivo*, from infusion to tumor clearance and long-term immunosurveillance. The acronym DIAL stands for Distribution, Infiltration, Accumulation, and Longevity, compartmentalizing the systemic attributes of gene-modified cellular therapy and providing a platform for optimization with the ultimate goal of improving therapeutic efficacy. This review will discuss both existent and emerging examples of DIAL characterization in mouse models, as well as opportunities for future development and optimization.

## 1 Introduction

Mouse models are routinely used in the preclinical characterization of gene-modified adoptive cellular immunotherapy candidates for clinical development. Adoptive transfer studies in mice, initially in the context of allograft transfer, underpin our understanding of T cell function, trafficking, and therapeutic potential (1–3). The first human studies of gene-modified T cells occurred in the 1980s (4), where a neomycin resistance cassette was retrovirally transduced into tumor-infiltrating lymphocytes (TIL) from patients with melanoma before infusion into patients. These pioneering studies demonstrated the ability of autologous lymphocytes transferred into patients, along with interleukin-2 (IL-2) treatment, to persist for months. The persistence of the transgene was determined through a PCR-based detection assay, which enabled detection of the adoptively transferred cells. Prior to this landmark human clinical study, a similar approach employing TIL therapy was successfully implemented in mice, resulting in complete regression of syngeneic murine tumors (5). Thus, the mouse study served as a valuable early model for investigating the distribution and function of adoptively transferred tumor-infiltrating lymphocytes.

The development of gene-modified chimeric T-cell receptors that enhance T cell function toward tumor cells in an MHC-independent manner was first described by Eshhar and colleagues in 1989 (6). These chimeric receptors, termed immunoglobulin T-cell receptor (TCR) chimeras, combined an extracellular antibody-like antigen binding molecule with a TCR and an intracellular CD3 zeta (CD3ζ) signaling domain. While effective *in vitro*, these initial designs lacked the co-stimulatory framework that enabled enhanced T-cell function and persistence *in vivo*.

In 2002, the “second generation” chimeric antigen receptor (CAR) was introduced, addressing the limitations of the previous design (7). This evolved design enabled transduced T cells to produce high levels of endogenous IL-2, supporting the maintenance of cytotoxic function and persistence. Over the years, multiple second-generation CAR-T were developed, incorporating co-stimulatory domains such as CD28 or 4-1BB (8). These designs differed from the first-generation constructs by their ability to drive T cell expansion in the presence of continual antigen exposure (9). The functional improvement was further demonstrated by the requirement of co-stimulation for tumor regression in mouse models of ALL (10, 11).

A few years later, the second-generation CAR-T design was reduced to practice with the first evidence of CAR-T-mediated tumor responses in human clinical trials in advanced follicular lymphoma (12). Subsequent clinical trials led to the development of second generation CAR-T therapy targeting CD19, resulting in the approval of tisagenlecleucel and axicabtagene ciloleucel for the treatment of refractory lymphoma and pediatric leukemia. Thus, the preclinical validation of second-generation CAR-T in mice informed the subsequent clinical development and approval of gene-modified lymphocyte (GML) therapy.

In parallel with second-generation CAR-T, transgenic T-cell receptor (TCR-T) therapy gained momentum as another genetically modified approach for redirecting potent T-cell responses in solid tumors. Building upon the initial success of TIL therapy, Rosenberg et al. engineered autologous gene-modified TCR-T against the melanosome antigen MART-1 (13). These modified TCR-T cells demonstrated persistence and induced tumor regressions in a limited number of patients. The development of the PMEL-1 mouse model, targeting another prevalent melanosome antigen in mice, provided strong preclinical evidence. In this model, transgenic PMEL-1 (gp100) directed T cells were transferred into mice bearing melanotic, poorly immunogenic B16 tumors (14). In both preclinical and clinical settings, tumor regression and persistence of the TCR-T were observed, accompanied by autoimmune responses such as vitiligo, which were observed in both mice and humans (15, 16).

The future development of therapies against HLA-restricted cancer-testis (CT) antigens including NY-ESO-1, PRAME, MAGE-A3 and MAGE-A4, among others, showcased additional opportunities for the efficacy of TCR-T against solid tumors. However, the development of a fully analogous mouse model posed challenges due to the lack of homology, expression and/or cross-reactivity of TCRs with mouse peptide-MHC (pMHC). In most cases, representative antigens with known high-affinity CD8 TCRs such as PMEL-1, tyrosinase-related protein 1 (TRP1) or ovalbumin (OVA/OT-I) were used as proxies for human TCR-T.

The process of preclinical validation for GML is constantly evolving, and new guidance is emerging (17). However, the primary objective of preclinical testing remains consistent: to confirm the biological activity, mechanism of action, and preclinical safety of the therapy before its use in patients.

While animal models, particularly severely immunocompromised mice, are commonly employed for CAR-T validation, the current approach often focuses on assessing fundamental attributes such as dose-dependence, on-target on-tumor activity, and the absence of uncontrolled proliferation and toxicity. While these attributes validate the functionality of the transgene and the manufacturing process, they overlook several critical aspects that should be considered when designing GML as therapeutics for oncology.

These aspects include modeling on-target, off-tumor toxicity and exhaustion, understanding the impact of lymphodepleting chemotherapy (LDC) on toxicity or cell expansion, evaluating tumor heterogeneity and antigen escape, evaluating the tumor microenvironment (TME) with its soluble factors and immune suppressor cells, overcoming barriers to infiltration, exploring the consequences (both positive and negative) of long-term expansion and persistence, and evaluating toxicities such as cytokine-release syndrome (CRS) and immune effector-cell-associated neurotoxicity syndrome (ICANS).

**Figure 1:**
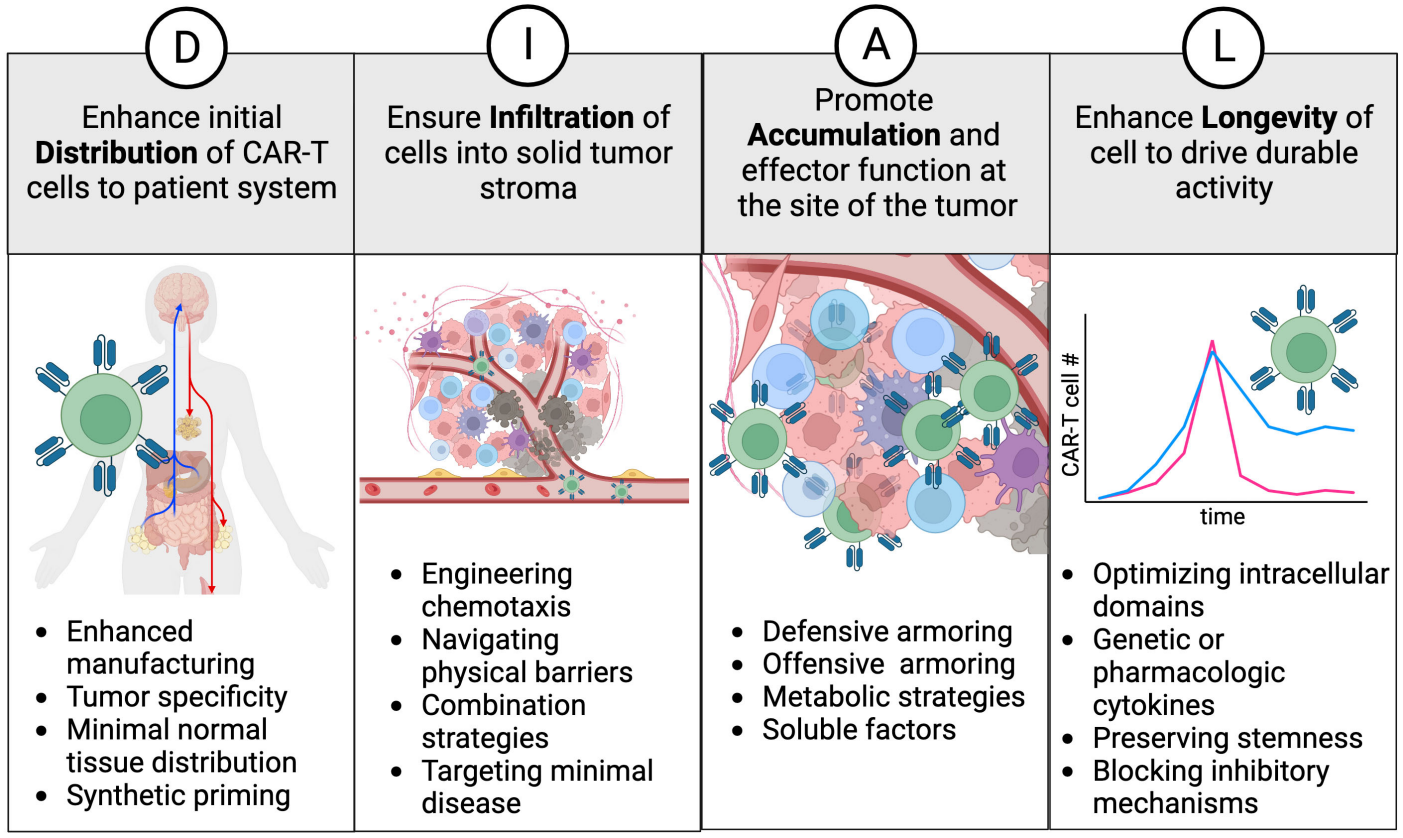
The DIAL framework. Distribution, infiltration, accumulation, and longevity are classified as key attributes and areas to focus on for enhancing cell therapy products. Examples of interventions are provided for each attribute and discussed in the text.

To characterize the cell therapy product and its design attributes more comprehensively, we have developed a framework called “DIAL”. This framework provides a holistic understanding of genetically engineered cellular immunotherapies *in vivo*, encompassing their journey from infusion to tumor regression and continued immunosurveillance. The acronym DIAL represents Distribution, Infiltration, Accumulation, and Longevity, which serve to compartmentalize the systemic attributes of gene modified cellular therapy (**Figure 1**). Additionally, DIAL serves as a platform for optimizing these attributes, with the ultimate goal of enhancing overall therapeutic efficacy. This review will discuss both existing and emerging examples of DIAL optimization, their characterization in mouse models, and potential avenues for future development. Given the rapidly evolving nature of this field, it is not feasible to cover each topic exhaustively with examples of each emerging technology. Instead, each section will focus on key examples, emphasizing areas for improvement and future advances.

## 2 Distribution

Optimal drug distribution refers to the efficient and rapid delivery of drug material to the desired site of action from the site of administration. The distribution of adoptively transferred lymphocytes for cellular therapy is analogous to pharmacologic distribution of biologics and small molecules, with certain key distinctions.

As donor-derived “living drugs”, infused GML exhibit a distribution pattern similar to endogenous lymphocytes (18–20). Following intravenous infusion, T-lymphocytes follow the vascular circulation, extravasate into tissues, diffuse into target sites, and recirculate through uptake in lymphatic tissue via high endothelial venules, ultimately returning to the vasculature through either the thoracic duct or vascular circuits (21). While the initial use of CAR-T focused on treating of B cell malignancies in the periphery it is important to note that GML also can distribute in compacted tissues and encounter their cognate antigens. This interaction allows them to receive survival and proliferation signals, promoting their expansion.

The expansion phase of GML products induced by antigen encounter sets adoptive cell therapy (ACT) apart from other therapeutic approaches. During this phase, the levels of the therapeutic cells (measured by the presence of CAR or TCR-positive cell by flow cytometry or gene copy number by PCR) increase relative to the infusion levels. The peak and duration of this elevation depends on the antigenic burden and the microenvironmental context of antigen encounter, both of which influence the extent of proliferation and expansion. Over time, the infused cells undergo apoptosis in various tissues. The initial phase of decay occurs rapidly after the initial expansion, while the second phase of decay proceeds gradually over weeks to months. This gradual decline may be attributed to factors such as loss of stimulation, activation-induced cell death, and exhaustion (22).

The composition of cell therapy infusion products plays a pivotal role in determining their distribution. When accounting for effector, memory and naïve subsets, both rodents and human exhibit a similar distribution pattern for ACT (21, 23–25). Factors such as cell size and the presence of receptors that govern lymphatic tropism (such as L-selectin and CCR7) can influence the survival and recirculation of cells post-infusion. Further, the relative composition of these subsets can impact the proliferative and metabolic potential of the transferred lymphocytes.

In the case of commonly used laboratory mice (Mus musculus), variations in CAR-T and TCR-T distribution may be attributed to factors like strain differences, extent of immune competence, and discrepancies in housing and sourcing facilities. Nonetheless, working with laboratory mice generally offers the advantage of predictability and consistency in experimental outcomes.

### 2.1 Distribution – cellular manufacturing

The ex vivo manipulation and expansion of GML, often referred to as cellular manufacturing, represents the initial stage where subsequent distribution patterns can be influenced. The manufacturing process involves complex interplay of factors that dictate the cellular kinetics (CK) of the grafted cells, including post-infusion cell survival, tissue infiltration, antigen-induced expansion, recirculation through the lymphatics into the bloodstream, terminal differentiation, and elimination.

Early T cell manufacturing processes focused on scaling up large numbers of cells for infusion into patients who would subsequently receive supportive doses of IL-2 (26, 27). In comparison, CAR-T therapy had key distinctions, requiring fewer cells for infusion and demonstrating the ability to persist without patient-administered IL-2. However, many of the principles established with TIL therapy, such as T cell activation though their TCR using anti-CD3 antibodies or antigen-presenting cells, and expansion in the presence of moderately high (>100 IU/mL) levels of IL-2 for 1-2 weeks, were initially maintained in GML protocols.

However, it was observed that sustained expansion in IL-2 could lead to cell exhaustion and limited persistence *in vivo* (28). Consequently, various protocol modifications were adopted in cellular manufacturing to preserve the fitness and persistence of the transferred cells post-infusion (29, 30).

In the years following the development of the initial CD19 CAR-T therapy, numerous modifications to manufacturing protocols were explored. These included the use of CD3/CD28 beads and alternative cytokines such as IL-7, IL-15, and IL-21, which promoted a stem cell memory phenotype (31–33). Additional enhancements involved incorporating small molecules into expansion media to further boost memory characteristics, promote persistence, and increase anti-tumor activity (34–39). The results demonstrated that process modifications preserving a memory cell phenotype enabled enhanced persistence, as determined by factors such as proliferative potential, resistance to stress (e.g., serial antigen stimulation or low cell dosing), and improved distribution to lymphatics and secondary lymphoid organs, facilitating recirculation (40).

Administration of high doses of CAR-T cells after prolonged manufacturing times and in conjunction with supportive IL-2 can lead to severe toxicities and lack of long-term durability, even when the target antigen is not present in normal tissues (41). Because of these potential issues, many of the recent advancements in autologous cellular manufacturing focus on preserving GML stemness as a means of improving potency and reducing toxicity, with a heightened focus on the T memory stem-cell (T_SCM_). This subset of memory T cells with self-renewal capacity was first identified in mice (34) and subsequently in human (28) and non-human primate (42). T_SCM_ have similar cell surface markers and metabolic potential compared to naïve T cells, and have an enhanced proliferative potential compared to other memory and effector subsets. For CAR-T, it is important to consider that recently activated naïve T cells are often not distinguished from native T_SCM_ cells and thus may differ in functional capacity and fate. There are a variety of ways to enhance the CAR- T_SCM_ fraction: mechanical enrichment of naïve/memory subsets (29, 43), addition of factors or cytokines to induce stemness (31, 37, 44), and shortening the manipulation of peripheral cells and reducing manufacturing time (45, 46). Although much of the modeling for CAR-T_SCM_ is performed using human cells in immunocompromised mice, the underlying principles can be applied to immunocompetent strains as well.

Murine T cell manufacturing processes are not optimal for translation to human CAR-T cell manufacturing. Mouse T cells exhibit many dissimilarities compared to their human counterparts. Additionally, available protocols for murine T cell transduction and expansion, as well as lymphodepletion, create disparities in comparison to humans.

To begin with, murine T cells do not expand ex vivo to the same extent as human T cells. They require different media and conditions for activation and do not consistently expand for greater than approximately 10-14 days. Furthermore, the source of cells differs between murine and human protocols. Murine protocols primarily utilize splenic T cells, whereas human protocols rely on peripheral blood T cells (47, 48).

Lastly, lymphodepletion in mouse typically involves low-dose (<5 Gy) whole-body irradiation, whereas lymphodepleting chemotherapy (LDC) is almost exclusively used in humans. These differing protocols can lead to variations in the dynamics of lymphopenic immune cell recovery and cytokine production, which are crucial for supporting the expansion of transferred cells and promoting memory phenotypes.

Despite these differences, genetically engineered fully murine model systems can still be utilized to highlight many of the advantages of generating stem-like T cells for ACT (49–55). In the future, the development and engineering of GML could benefit from a combination of both immunocompetent syngeneic murine and immunocompromised human xenograft model systems. This approach would provide a more comprehensive understanding of the various factors required to achieve optimal cell distribution.

### 2.2 Distribution – choice of antigen

**Figure 2:**
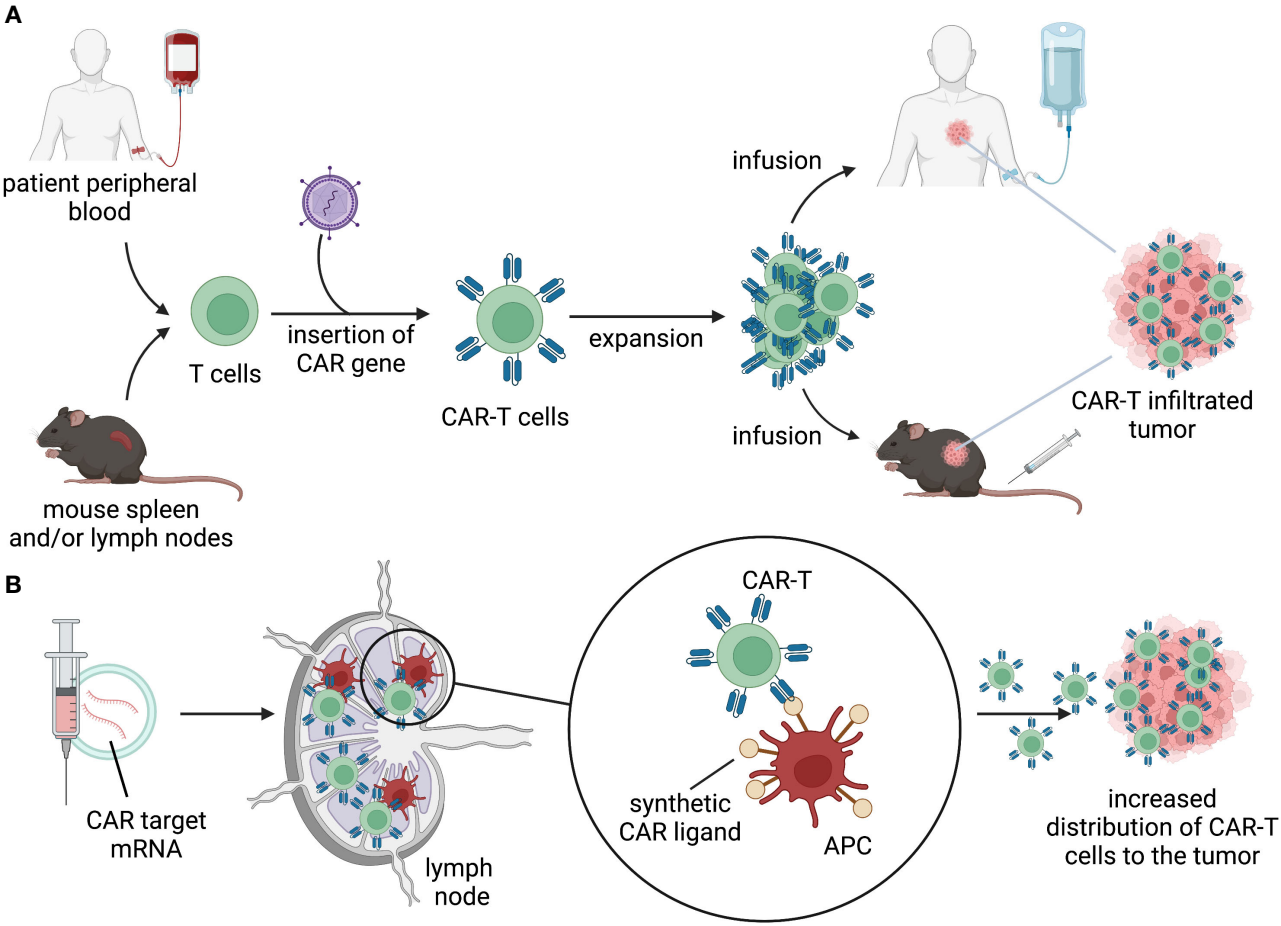
Distribution. **(A)** T cells isolated from patient’s peripheral blood or mouse lymphoid organs are engineered to express a CAR. CAR-T cells are expanded ex vivo and infused back into the patient or in tumor-bearing mice. Upon infusion, CAR-T cells distribute through the body, migrate to the tumor, and kill target-expressing tumor cells. **(B)** delivery of mRNA-encoding CAR ligands using lipid nanoparticles (LNPs) to lymph nodes results in expression of the target by APC. Target-expressing APC prime and activate CAR-T cells, allowing for expansion and migration to the tumor.

The choice of antigen and context of antigen encounter will influence the initial distribution of the gene-modified cells. After intravenous infusion, cell therapy products follows a pattern of distribution that involves vascular circulation, extravasation, diffusion into tissue, and recirculation primarily through lymphatics and vascular circuits. GML can distribute in tissues and encounter their cognate antigens in the tumor, triggering survival and proliferation signals that allow for expansion and continued distribution (**Figure 2A**). Different types of lymphocytes may have varying tendencies to distribute to tissues and lymphatics.

In cases where antigen is readily accessible in blood-based tumors, the process of expansion and distribution is facilitated. However, when the antigen is confined to poorly vascularized or compacted tissues, both the accessibility and the context of antigen encounter are compromised. Non-genetically modified lymphocytes, particularly T cells, have the capability to produce cytokines and transcription factors that promote proliferation and antigen-induced expansion. GML, with their expressed receptors, further enhance antigen-induced expansion to exaggerated levels, which can be augmented by preceding lymphodepletion. It is important to note that the differences between mouse and human lymphodepletion, as discussed earlier, can also lead to discrepancies in interpreting preclinical models when evaluating antigen-specific expansion in humans.

Antigens that exhibit a tumor-specific expression profile facilitate confined and intended cell expansion. On the other hand, more promiscuous antigens increase the risk of “off-tumor” toxicities and promote effector differentiation, which can lead to eventual dysfunction. When target-directed activity induces both direct cytotoxicity and paracrine effects through cytokine production, widespread antigen expression outside the tumor can exacerbate systemic inflammation. This, in turn, hampers the cells’ ability to infiltrate the tumor efficiently. This phenomenon is commonly referred to as on-target, off-tumor toxicity. However, promiscuous antigen expression can also impede cell expansion and distribution by diverting cells into non-tumor tissues, effectively excluding a portion of the transferred GML from their intended target.

The ideal antigen for tumor targeting would be uniquely expressed in the tumor and absent in normal tissues. However, for CAR-T therapy, such antigens are rare, with only a few examples like EGFRvIII and aberrantly glycosylated protein glycoforms such as Muc1 falling into this category (56, 57). Most CAR targets show a preference for tumor expression but are also detected in normal tissues. The most successful CAR targets have been observed in B cell malignancies, where normal expression occurs in cells that are apparently dispensable. Another wave of CAR targets is emerging, with lower levels of normal tissue expression but not complete absence. These targets aim to strike a balance between effective tumor targeting and minimizing off-target toxicities. It is challenging to achieve complete avoidance of target engagement in normal tissues, so efforts are focused on selecting targets with reduced normal tissue expression to mitigate potential toxicity while still enabling successful tumor targeting.

This expanded repertoire of CAR targets opens up new possibilities for successful immunotherapy beyond B cell malignancies, where targets expressed in dispensable tissues have shown promising outcomes. Preclinical mouse models provide certain examples where on-target, off-tumor toxicity can be monitored. However, it’s important to note that these models often involve immunocompromised mice using mouse cross-reactive single-chain variable fragments (scF*vs*) or transgenic approaches to introduce the human antigen.

For instance, one study showed that a low-affinity Her2 CAR-T specifically targeted Her2-expressing tumors while avoiding accumulation in the mouse liver, where Her2 was artificially expressed using adenovirus (58). In another example, an affinity-tuned, mouse cross-reactive GPC3 CAR-T was able to avoid lethal toxicity against GPC3 expressed in the mouse lung and induce regression of human hepatocellular carcinoma tumors (59). However, caution must be exercised when interpreting off-tumor toxicities in mice, as some results may not align with human studies. For instance, a high-affinity GD2 CAR-T demonstrated lethal CNS toxicity in a mouse model of neuroblastoma (60), whereas human clinical trials using a similar GD2-targeted CAR-T via intravenous and intracerebroventricular therapy showed good tolerability without signs of off-tumor CNS toxicities (61).

In contrast to CAR therapy, GML TCR therapy offers access to certain tumor-specific targets, particularly due to its ability to recognize intracellular proteins presented as peptides in the context of the major histocompatibility complex (pMHC). These targets primarily fall into the categories of cancer-testis antigens, tumor-viral antigens, and mutant neoantigens. The presence of these unique tumor-specific targets presents opportunities for precise and personalized immunotherapy approaches.

GML TCR therapy allows for the targeting of antigens that are specifically expressed by tumor cells, offering potential advantages in terms of enhanced tumor specificity and reduced toxicity compared to CAR therapy. This approach broadens the range of tumor-specific antigens that can be targeted, expanding the possibilities for developing effective treatments against various types of cancer. However, achieving broad targeting will necessitate the rapid development of personalized TCR-T therapies (62), which cannot be practically modeled in mice.

For the majority of the aforementioned targets, fully mouse surrogate CAR/TCR systems, transgenic mice, or cross-reactive mouse antibodies are not typically used in preclinical studies. While on-tumor activity can be measured, there remains a significant gap in the investigation of off-tumor inflammation and toxicity. Consequently, examples of off-tumor engagement by GML are predominantly observed in human clinical trials, and the overall implications for cell survival, distribution, and toxicity in these scenarios remain poorly understood.

Furthermore, it’s worth noting that many GML TCR therapy approaches are often combined with interleukin-2 (IL-2) to enhance cellular expansion and persistence. However, the use of IL-2 in these therapies brings its own considerations and potential dose-limiting toxicities. These toxicities may include vascular leak syndrome, hypotension, and renal dysfunction. Modeling IL-2 toxicity in mice is challenging, primarily because mice can tolerate high levels of IL-2 without experiencing the dose-limiting toxicities observed in humans.

A recent advancement in GML engineering is the application of combinatorial antigen targeting technology. This approach aims to more precisely target tumor tissues while avoiding off-tumor inflammation and promoting optimal GML distribution. However, it is important to note that most logic-gated CAR-T systems have only been demonstrated at the preclinical level in mouse models.

Logic gating approaches typically involve two distinct binding events and can fall into different categories:

1. OR gating: Both antigen-binding fragments can simultaneously engage with the target and trigger signaling. This can occur through tandem engagement, where both antigen-binding molecules are joined and signal through a shared intracellular domain (ICD), or through the expression of separate CARs in a bicistronic format.
2. AND gating: Both antigen-binding fragments are required to engage the target in order to initiate signaling.
3. ON gating: The CAR is not expressed or fully functional until the addition of a second pharmacologic agent, which can be a small molecule, protein, or antibody-like drug. “Universal CARs” also fall in this category.
4. NOT gating: The second antigen-binding fragment can disable or “turn off” the signaling initiated by the first antigen binder, typically expressed in normal tissues.
5. IF … THEN gating: The first antigen binder drives signaling through a secondary transcription factor, leading to the expression of the second protein, which can be a fully competent CAR molecule.

While “OR” gated CAR-T, used in multiple dual and tandem formats, is being evaluated in clinical trials for hematologic malignancies, most logic-gating approaches have only been described and validated in preclinical studies in mice (63–66). Since most logic-gating strategies require two separate events to initiate signaling, they may have limitations such as leakiness or a loss of precise temporal control. For a more detailed discussion on the benefits and challenges associated with various logic-gating approaches to mitigate on-tumor, off-target toxicity, a recent review provides in-depth coverage (67).

An innovative approach aims to address some of the challenges faced by other technologies by combining distinct antigen binding domains with mutually dependent signaling domains downstream of the TCR complex. Researchers proposed coupling the intracellular domains (ICDs) of SLP76 and LAT to single-chain variable fragment (scFv) domains that recognize different antigens. By engineering mutations that prevent simultaneous binding of the Grb2 family member GADS, this approach creates a tightly regulated logic gate that has the potential to enable tumor-specific expansion of dual CAR-T cells (68).

The SLP76/LAT logic gate was validated using a commonly used xenograft model of acute lymphoblastic leukemia (NALM6) in NSG mice, where the tumor cells expressed ROR1 and CD19 antigens as dual targets. Encouragingly, this approach demonstrated an improved balance between efficacy and off-tumor toxicity compared to traditional “split CAR” and “Syn-NOTCH” logic-gating strategies. The development of technologies that provide more precise control over dual antigen targeting in the future may not only help in managing off-tumor toxicity but also enhance the distribution properties of CAR targeting molecules.

This advancement opens up possibilities for the exploration of dual-antigen-defined tumor types. It offers the potential for improved efficacy by combining the favorable attributes of TCR GML (tumor specificity) and CAR GML (targeting broader indications).

### 2.3 Distribution – cell priming strategies

To enhance GML expansion, strategies have been explored to mimic peripheral priming mechanisms in secondary lymphoid organs (SLO) by targeting oxygen, nutrient, and co-stimulatory ligand-accessible regions of the body. Initially, the concept of enhancing adoptive T cell therapy involved using viral-specific T cells as a platform for tumor-directed CAR or TCR transgenes. These priming responses can be engaged passively, through endogenous viral antigen presentation, or actively, via vaccination against the viral antigen (69, 70). In recent approaches, *in vitro* priming of CAR-T cells with oncolytic viruses through their native TCR has been combined with *in vivo* boosting, leading to impressive responses in preclinical models of melanoma and glioma (71).

Despite the early enthusiasm and adoption of viral-specific T cells as a CAR platform, and the development of innovative approaches to prime peripheral expansion of CAR-T, only a limited number of human clinical trials have been initiated using these approaches. It’s important to note that most viral-specific T cells are being explored as an allogeneic platform due to their reduced potential for alloreactivity (NCT04288726, NCT03740256, NCT01475058, NCT01430390). In addition to viral-specific T cells, other lymphocyte lineages such as NK cells, iNKT cells, γδ T cells, and MR1 T cells that can recognize non-MHC antigens in tumors may also be considered as GML platforms (72–75). While these alternative ACT platforms have the ability to naturally expand in the presence of their cognate ligands, similar to viral-specific T cell priming, and show potent responses in mouse models, it remains to be seen if they exhibit similar expansion and distribution potential to αβ CAR-T cells in human patients.

Recent synthetic approaches to enhance CAR-T expansion include “CAR vaccine” strategies, which involve the delivery of mRNA-encoded CAR ligands using lipid nanoparticles (LNPs) to lymph nodes (**Figure 2B**). This allows CAR-T cells to undergo *in vivo* expansion and stimulation in lymphatic regions. The CARVac strategy demonstrated that LNP-mediated delivery of the CLDN6 antigen to antigen-presenting cells (APCs) in the lymphoid compartments of mice could promote robust expansion of CLDN6 CAR-T cells and improved anti-tumor efficacy (76). Another approach involved *in vivo* expression of “amphiphile ligands” that could be delivered by LNPs and inserted into the membrane of APCs to present cognate ligands, facilitating GML proliferation (77, 78). Interestingly, neither of these approaches resulted in substantial elimination of the CAR-antigen expressing APCs. Clinical testing of CARVac is underway to enhance CAR-T expansion against CLDN6-positive solid tumors (NCT04503278). Although data generated in mouse models demonstrated a significant increase in expansion and improved anti-tumor efficacy, data from human trials are still in the early stages and inconclusive regarding the priming benefits of the “CAR vaccine” approach. Nonetheless, these and future strategies to safely enhance peripheral CAR-T expansion can be effectively modeled in mice and have the potential to drive GML activity using lower infused doses or lymphodepletion-free regimens.

## 3 Infiltration

Following successful systemic distribution, transferred immune cells may encounter various physical and chemical barriers as they navigate towards tumor-associated antigens and exert their cytotoxic function. In the case of hematologic malignancies, GML may not require extensive infiltration to encounter antigens. However, this is not the case for the majority of carcinomas. Fortunately, immune cells, such as T cells, possess receptors that facilitate extravasation from the vasculature into inflamed tissues and further penetration into the extracellular matrix (ECM) based on chemokine receptor expression, cytokines, and chemical gradients (79). While most CXC and CC chemokine receptors are already expressed on peripheral T cells at various activation stages (80), there are instances where enforced overexpression of these receptors enhances GML’s ability to access tumors by leveraging native chemical gradients.

### 3.1 Infiltration – engineering chemotaxis

**Figure 3:**
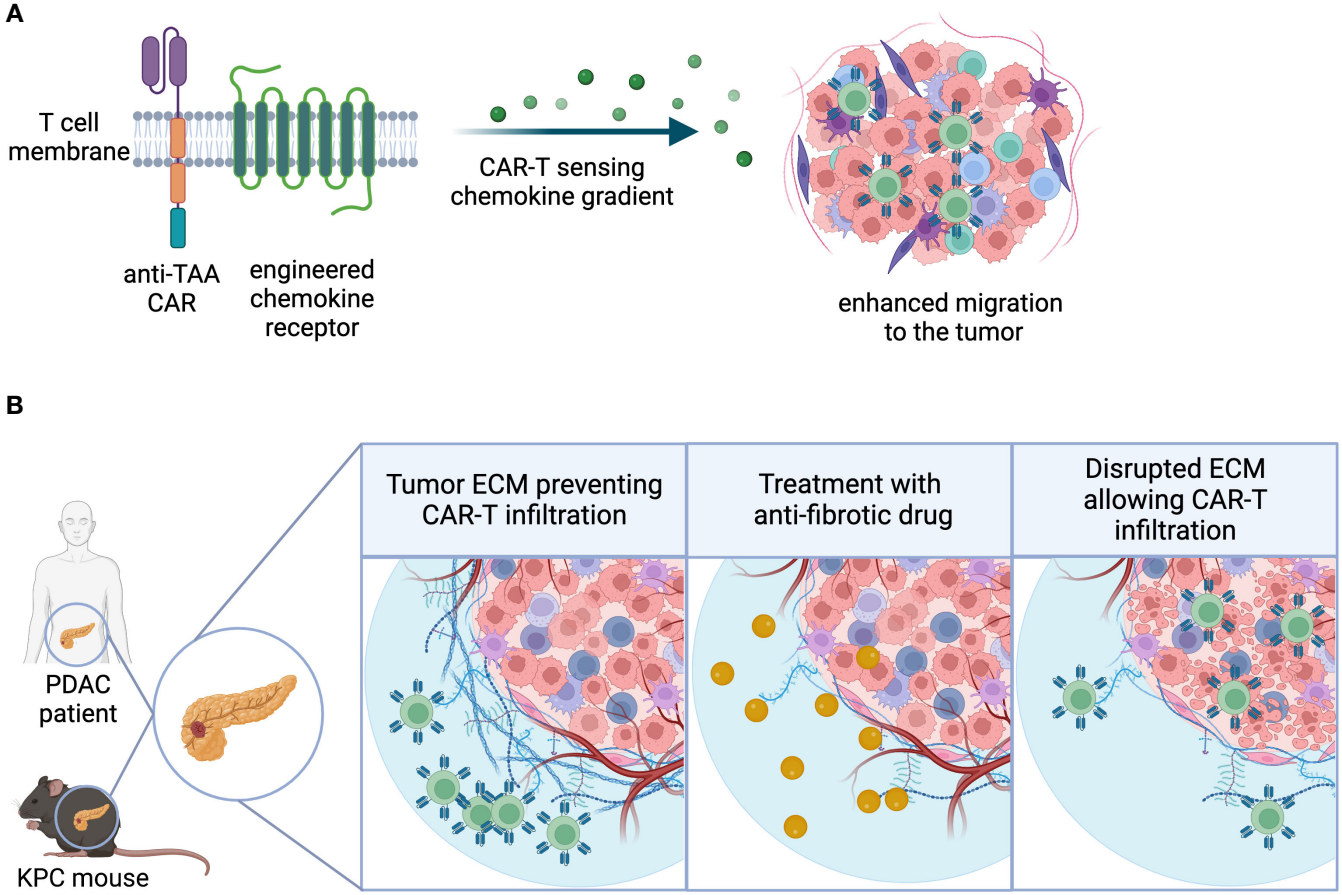
Infiltration. **(A)** Chemokine receptors can be engineered into CAR-T cells, enhancing the sensitivity to chemokine gradients and ultimately migration and infiltration to the tumor site. **(B)** Extracellular matrix-(ECM) rich tumor types physically restrict lymphocytic infiltration, creating an immune-excluded TME. Use of combination agents that directly or indirectly target tumor fibrosis may facilitate GML infiltration into the TME.

One important aspect of successful infiltration is the ability of T lymphocytes to undergo extravasation and chemotaxis towards sites of inflammation. Naturally circulating T cells acquire this capacity as a result of effector differentiation following antigen recognition and priming in secondary lymphoid organs (SLO). As described earlier, there are various methods to enhance GML priming using synthetic and natural mechanisms. Additionally, receptor overexpression in GML has been shown to augment their infiltration capacity. Some approaches focus on tumor-specific cues to modify chemotactic receptor expression, while others aim to increase lymphoid-homing capacity, recirculation, and survival in a more general manner, thereby enhancing anti-tumor function (81). Proof-of-mechanism for tumor-specific strategies primarily comes from engineered preclinical mouse models and xenograft model systems, where overexpression of the chemokine receptor can influence the infiltration and accumulation of CAR-T cells compared to their unmodified counterparts.Chemokine receptor overexpression in GML has been utilized to address potential trafficking deficiencies in solid tumors (**Figure 3A**). The strategic rationale behind these approaches is often similar:

1. Tumors are found to express sufficient levels of chemokine ligands, creating chemotactic gradients.
2. Peripheral T cells or endogenous tumor-infiltrating lymphocytes (TIL) show low levels or downregulation of the cognate or paired chemokine receptor(s).
3. Re-expression of the lost or downregulated receptor is shown to synthetically augment and/or restore infiltration in preclinical mouse models.

Examples of receptors engineered into GML include CCR2, CCR4, CXCR2, CXCR3, CXCR4, and CX3CR1 (82–88). Recent examples showed overexpression of CXCR2 enhanced infiltration of CAR-T hepatocellular carcinoma xenografts where the tumors were shown to express high levels of CXCR2 ligands including CCL2, CXCL1, CXCL2, and CXCL5 (89); CXCR1 overexpression enhanced T and NK CAR cells to migrate into ovarian or pancreatic tumors (90, 91) and CCR2 transduction into MSLN CAR-T cells enhanced migration into CCL2 positive malignant pleural mesotheliomas (86, 92). Although some of these examples exploit chemotactic gradients that may be generally expressed in inflamed tissues rather than specifically in tumors, a recent study used a tumor-specific strategy by forcing overexpression of the chemokine receptor CXCR6 on mesothelin-targeted CAR-T cells. This approach led to enhanced regression of pancreatic tumor xenografts. CXCR6, which is normally lowly expressed on peripheral blood T cells, is naturally attracted to its counterpart ligand CXCL16. In this case, CXCL16 was found to be highly expressed by malignant pancreatic epithelial cells, making it an attractive tumor-specific strategy (93).

Other strategies have been explored to improve infiltration and survival of transferred ACT without directly targeting the tumor. Some of these strategies involve factors such as IL-7/CCL19 or L-selectin overexpression to increase tumor penetration of ACT (81, 94). Notably, L-selectin overexpression in mice allowed for equivalent tumor infiltration and improved anti-tumor function of CAR-T cells by decreasing the activation threshold of the transferred cells, contrary to the presumed improvement in lymphoid organ homing. While these strategies have shown therapeutic merit in preclinical models, few have been validated in human clinical trials. It remains to be seen whether these strategies can enhance the success of GML-based solid tumor therapy.

A recent report suggested that ex vivo expansion of CAR-T cells in the presence of TGF-β could significantly enhance tumor infiltration and efficacy in xenograft tumor models (95). This approach aimed to epigenetically rewire peripheral blood T cells toward a resident memory T cell phenotype (T_RM_), which would enhance accumulation and promote stemness of the transferred CAR-T cells targeting MSLN. In CAR-T_RM_ treated, regressing xenograft tumors, there was a marked increase in CAR-T cell infiltration, and depletion of CD103+ cells diminished the benefits of the so-called CAR-T_RM_ cells. Interestingly, overexpression of the T_RM_ transcription factor RUNX3, which had been used previously to enhance TRM expansion in murine tumor models, did not phenocopy the CAR-T_RM_ cells (96). This expansion strategy represented the first attempt to enhance CAR-T infiltration in murine models using ex vivo process engineering. Intriguingly, a similar protocol was used in human clinical trials to enhance neoantigen-directed TCR-T cells targeting mutant KRAS G12D pMHC (97). In this clinical trial, a TGF-β-based ex vivo expansion protocol was employed to enhance TCR-T cell infiltration in pancreatic ductal adenocarcinoma, leading to a dramatic reduction of lung lesions and a durable clinical response in one patient.

Another strategy to improve GML infiltration involves introducing chemotactic factors or cytokines into tumor-targeted therapeutics, such as engineered oncolytic viruses (OVs). Studies using mouse models demonstrated that intra-tumoral injection of adenovirus engineered to produce IL-15 and RANTES could promote GD2 CAR-T cell infiltration into solid tumors in immunocompromised mice (98), and this approach is also being explored in human clinical trials (NCT03740256). Subsequent efforts showed that intra-tumoral injection of OVs, such as vaccinia, could promote T cell infiltration by producing high systemic levels of CXCL11 (99). Other combinations propose introducing cytokine or immune checkpoint blockade (ICB) payloads into OVs, which, together with the oncolytic activity of the virus, can enhance CAR-T cell infiltration and survival in preclinical models of solid tumors (100–102). Unexpectedly, OV-mediated introduction of intra-tumoral Type 1 interferon (IFNβ) was shown to drive CAR-T cell apoptosis, which was prevented by deleting IFNAR1 in the engineered cells (103). Given that many OVs naturally induce the production of Type I interferon, a deeper understanding of the potential benefits, pitfalls, timing, and sequencing of combination OV and CAR-T therapy is warranted (104). The combination of OV and CAR-T therapy for solid tumors is a subject of ongoing research and a separate review (105).

### 3.2 Infiltration – navigating physical barriers

In addition to challenges posed by suboptimal chemotactic gradients, solid tumors often create physical barriers that promote immune exclusion. Immune exclusion can be caused by immune or non-immune stroma in the TME, which may impede immune cell infiltration. The next section of this review will delve into the discussion of soluble and metabolic factors that contribute to immune exclusion. This section will primarily focus on tumors characterized by fibrogenic stromal cells, also known as cancer-associated fibroblasts (CAFs), which produce collagen-containing matrices that physically restrict lymphocytic infiltration. However, it is important to note that certain human tumors, such as pancreatic ductal adenocarcinoma, possess extensive stroma that cannot be accurately modeled in preclinical species. Despite this limitation, preclinical strategies have been developed to counteract non-immune stromal cells using CAR-T cells, either by directly eliminating CAFs or indirectly targeting stromal-derived barriers. In addition, use of combination agents that directly or indirectly target tumor fibrosis may represent an alternative strategy to improve GML infiltration into extracellular matrix-(ECM) rich tumor types (**Figure 3B**).

Developing strategies to target the extracellular matrix (ECM) presents multiple challenges. One of the key challenges is the development and validation of preclinical models that accurately replicate CAF-containing fibrogenic stroma observed in pancreatic, colon, hepatocellular, gastric, esophageal, head and neck, cervical, and breast cancers. While mouse models can partially capture certain aspects of fibrotic tumors, such as modeling the increased interstitial fluid pressure induced by fibrosis (106), they are likely to fall short in simulating the complexity of combining fibrosis-targeting agents with GML. Additionally, CAFs, like other components of the immune stroma, are heterogeneous and play diverse roles in cancer, some of which may not be adequately modeled in mice. Furthermore, fibrogenic stroma in humans often develops in the pre-metastatic niche, resulting from the recruitment of factors from the bone marrow and facilitating vascular access before tumor establishment. Most of the strategies mentioned above have been described using cell line-derived xenograft (CDX) or genetically engineered mouse models (GEMM), which rarely fully replicate the extent of stiff, fibrotic ECM seen in cancer patients.

Strategies encoded in GML to target fibrosis include directly eliminating fibrogenic cells in the tumor. One of the prominent targets on CAFs is the fibroblast-associated protein (FAP) (107–109). Murine reactive FAP CAR-T cells have shown effectiveness in multiple preclinical models, but potential toxicity concerns arise due to the depletion of FAP-expressing bone marrow stromal cells (110, 111). There have been limited opportunities to test this concept in human clinical trials, but FAP CAR-T cells appear to be tolerated at subtherapeutic doses (NCT01722149). Another genetic strategy to enhance access to ECM-rich tumors involves engineering CAR-T cells to secrete ECM-degrading enzymes, such as hyaluronidase or heparinase (112, 113). In both preclinical examples, the encoded and secreted enzymes did not interfere with CAR-T cell function and effectively degraded the matrix in mouse xenograft tumor models. Although these efforts have shown benefits in various murine models, there is limited proof-of-concept evidence from human clinical trials regarding ECM degradation and/or fibroblast targeting.

Myeloid cells can also impede GML infiltration into tumors. Targeting tumor-associated macrophages (TAMs) and myeloid-derived suppressor cells (MDSCs) broadly for depletion can be challenging due to potential toxicities. However, recent studies suggest that certain receptors may serve as tumor-specific targets, allowing for safer targeting of these suppressive cell types. For TAMs, targeting the folate receptor β (FRβ) with a CAR in syngeneic mouse models was shown to be safe and promoted increased infiltration of MSLN CAR-T cells in multiple tumor models (114). MDSC targeting was facilitated by a DR5 agonist costimulatory CAR expressed on the cell surface of Mucin 1 (MUC1) CAR-T cells (115). This approach demonstrated improved anti-tumor activity in an MDSC-admixed tumor model, presumably through increased infiltration, although direct evidence was not provided. Interestingly, the concept of targeting MDSCs with DR5 agonist antibodies has been previously tested in mice and humans, showing promising results and offering a novel approach to enhance GML infiltration (116, 117).

### 3.3 Infiltration – combination strategies

An indirect approach to disrupt factors that suppress lymphocytic infiltration involves tumor debulking through surgery, chemotherapy, or radiation. However, these therapies can have a dual effect as radiation and chemotherapy-resistant tumors often develop fibrosis as a consequence of treatment (118). This fibrotic response can vary depending on the therapeutic regimen but is a common outcome due to oxidative stress in the TME. To address therapy-induced fibrosis, targeted therapies have been explored to inhibit cancer-associated fibroblasts (CAFs), the extracellular matrix (ECM), or overall tumor architecture.

In terms of targeting the ECM, small molecule inhibitors of lysyl oxidase (LOX) and LOX-blocking antibodies have shown impressive activity in a GEMM of breast cancer. However, these approaches have not yet demonstrated success in human clinical trials (119, 120). Other small molecule inhibitors such as fasudil (ROCK inhibitor) and defacitinib (FAK inhibitor) have been used to attenuate focal adhesion signaling in tumor cells, influencing ECM remodeling by reducing matrix metalloproteinase (MMP) deposition and the desmoplastic response, respectively (121). Both fasudil and defacitinib have shown improved survival in mouse models of pancreatic cancer (LSL-KrasG12D/+; LSL-Trp53R172H/+;Pdx-1-Cre), and early-stage clinical trials combining defacitinib with immune checkpoint inhibitors (ICI) have demonstrated stable disease with increased T cell infiltration in post-treatment biopsies. Although there are currently no planned clinical studies with fasudil, its potential remains to be explored. Recombinant hyaluronidase or pegylated recombinant hyaluronidase 20 (PEGPH20) have been tested in combination with chemotherapy for pancreatic ductal adenocarcinoma (PDAC) based on strong combination activity observed in KPC mouse models (106). PEGPH20 was able to reduce interstitial fluid pressure (IFP) in these models and improve the response to chemotherapy (gemcitabine + nab-paclitaxel). However, a randomized controlled Phase III trial of this combination did not demonstrate a significant benefit. The reasons why the KPC mouse model was not predictive of an improved combination response remain unclear. Another preclinical strategy involves targeting discoidin domain receptor 1 (DDR1) with neutralizing antibodies to disrupt collagen-containing stromal fibers (122). Targeting DDR1 to reduce collagen disposition enhanced the effects of chemotherapy and GML in orthotopic tumor models and mouse models of pancreatic cancer (KPC mice). To date, GML combinations with targeted inhibitors of ECM deposition have shown strong effects in multiple mouse models but have not translated successfully in clinical trials. This highlights the need to develop more predictive preclinical models of desmoplasia and matrix deposition to improve the infiltration of GML into tumors.

Administration of GML directly following chemotherapy may improve infiltration by exerting anti-fibrotic effects and shifting suppressive myeloid cells to a pro-inflammatory phenotype. Murad et al. demonstrated improved infiltration following chemotherapy in murine preclinical studies using cyclophosphamide prior to PSCA CAR-T administration in metastatic prostate and pancreatic cancer models (123). Interestingly, cyclophosphamide has been used to treat pulmonary fibrosis due to its lymphodepleting mechanism of action, which can suppress fibrotic inflammation (124). Alternative preconditioning therapies, such as fludarabine/cyclophosphamide (Flu/Cy) plus nab-paclitaxel (FNC), have been used in human clinical trials of CLDN18.2 CAR-T (125). These regimens, incorporating taxanes in addition to standard Flu/Cy, may improve GML efficacy by reducing tumor burden, conditioning the TME, and enhancing infiltration. However, there is a lack of preclinical mouse studies supporting the use of FNC preconditioning. The differences in metabolism of conventional chemotherapies between mice and humans make it challenging to model these combinations accurately, but exploring indication-specific regimens to improve responses should be considered.

Another potential approach to enhance GML infiltration involves intra-tumoral injection of surgically resected or debulked tumors to facilitate T cell-mediated clearance of minimal disease. Mouse models utilizing gel-embedded CAR-T have been described, demonstrating clearance of partially resected MSLN+ xenograft tumors (126). The fibrin gel-embedded MSLN CAR-T enabled clearance of tumor cells at the margin of surgical resection, minimizing on-target, off-tumor toxicity and complications associated with wound healing. The fibrin glue appeared to support CAR-T survival and infiltration into the tumor margin, clearing minimal residual tumor cells and preventing additional treatment-induced inflammation and fibrosis. A study to explore this approach in locally advanced breast cancer is planned.

## 4 Accumulation

**Figure 4:**
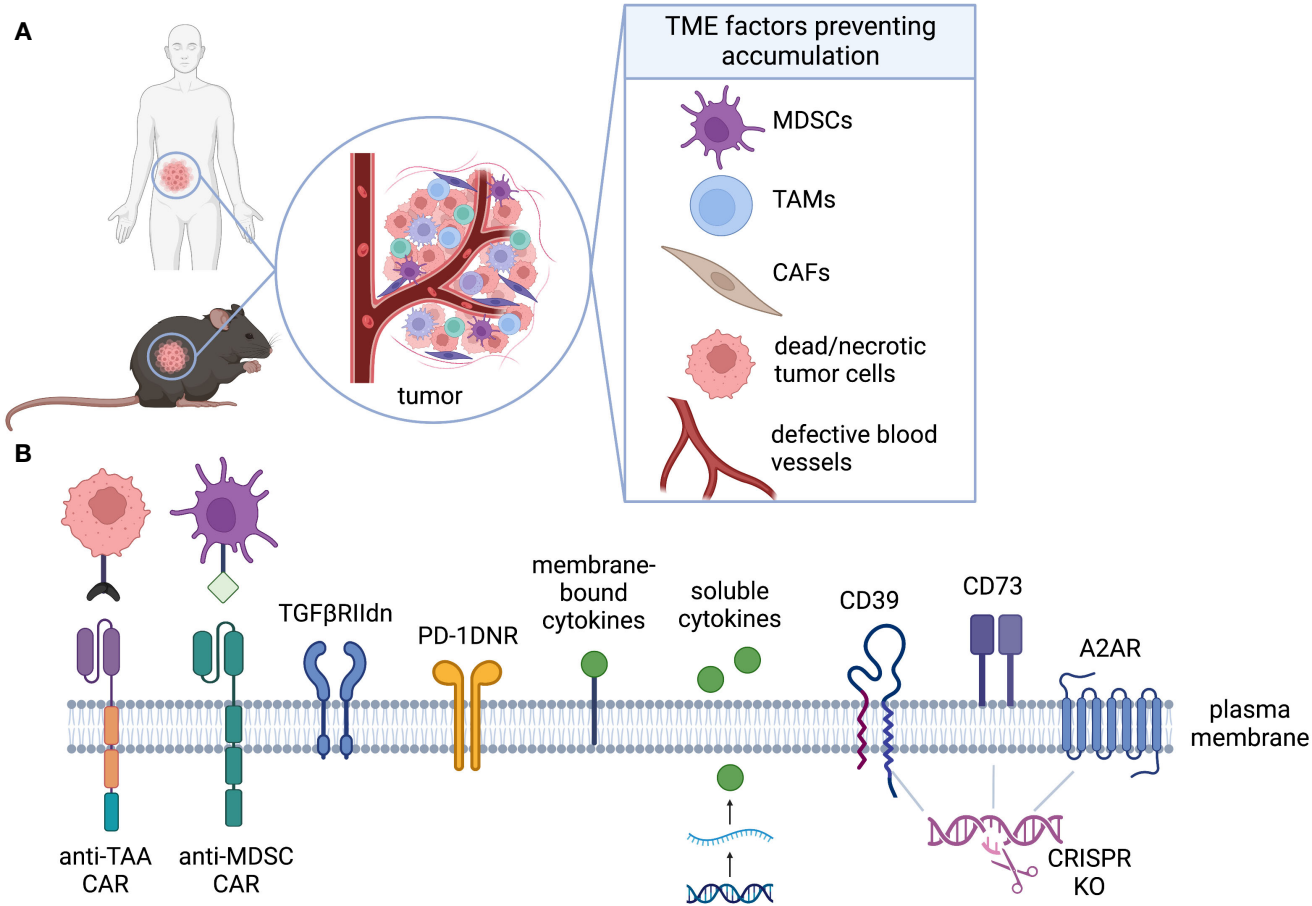
Accumulation. **(A)** The TME is rich of immunosuppressive factors that can inhibit CAR-T activation, proliferation, and accumulation even in presence of target antigen. **(B)** CAR-T can be engineered to alleviate distinct challenges of the TME. Armoring strategies can shield CAR-T cell, decreasing their sensitivity to inhibiting factors (TGFβ, PD-L1, adenosine), or can endow them with the ability to target immunosuppressive cells (anti-MDSCs).

In comparison to hematological malignancies, solid tumors present numerous challenges within the TME that hinder the activation and functionality of T cells, thereby limiting the efficacy of cell therapies (**Figure 4A**). Various factors within the TME can impede the successful accumulation and subsequent expansion of T cells, including metabolic properties (such as hypoxia, nutrient limitation, and ionic imbalance), the presence of suppressive cell types (such as TAMs, MDSCs, and Tregs), and the presence of suppressive cytokines or soluble factors (such as TGF-β and IL-10) (114, 127). Genetic engineering strategies for CAR-T cells aimed at counteracting the suppressive TME are commonly referred to as “armoring” approaches (128).

While there are diverse strategies to enhance the performance of CAR-T cells, a primary objective is to improve their accumulation within the TME. This can be achieved by enhancing their survival or promoting increased cell division rates. In this review, we classify accumulation strategies as either “defensive” or “offensive.” Defensive strategies aim to protect GML from factors that suppress their proliferation or survival, while offensive strategies involve the elimination of suppressive cells or factors to facilitate GML accumulation (**Figure 4B**). To provide preclinical validation, we will also assess the feasibility of modeling these strategies in mice.

### 4.1 Accumulation - defensive strategies

#### 4.1.1 Metabolism

T-cell metabolism plays a crucial role in determining both cell survival and the effectiveness of antitumor immune responses. However, understanding the intricate metabolic crosstalk between CAR-T cells and TME poses challenges for *in vitro* modeling. Therefore, the utilization of *in vivo* models becomes highly significant in dissecting the complex metabolic pathways involved in this interaction. Syngeneic models have emerged as valuable tools for investigating T-cell metabolism due to their flexibility in engineering specific immune subsets and accurately representing immune components within the TME.

Chang et al. utilized a sarcoma model expressing or lacking the spectrin-β2 antigen, which determines tumor rejection, to investigate T-cell fitness in different TMEs. Expanding upon these findings, the researchers further investigated T-cell metabolism using OT-I mice. Their groundbreaking work revealed a fundamental concept: the competition for glucose between CAR-T cells and the TME leads to reduced cytokine production and facilitates tumor growth (129). Additional insights into aerobic glycolysis-induced T-cell differentiation came from studies employing CD4^Cre^Ldha^fl/fl^ mice lacking LDHA in T cells. These studies demonstrated that aerobic glycolysis promotes effector T-cell differentiation, highlighting LDHA as a critical therapeutic target (130). Subsequently, transgenic PMEL-1 mice were used to explore the modulation of LDHA in cell therapy. This study demonstrated that the metabolic characteristics of antigen-specific T cells, along with their persistence, accumulation, and antitumor immunity, could be effectively modulated through the administration of exogenous cytokines (131).

The adjustment of T cells to hypoxic and nutrient-deprived tumors triggers a metabolic switch to glycolytic metabolism, primarily mediated by the transcription factor HIF-1α (132, 133). The critical role of HIF-1α in inducing T cell effector status has been demonstrated using HIF-1 or HIF-2α^fl/fl^ dlck^CRE^ mice (134). However, under chronic hypoxia, this metabolic switch might lead to terminal differentiation, anergy, and exhaustion. This is particularly relevant for transferred lymphocytes, which lack access to native priming mechanisms that can maintain memory cell persistence for extended periods. Therefore, rewiring the hypoxia response could potentially tailor T-cell differentiation, sustain persistence, and enhance antitumor responses in CAR-T cells.

Syngeneic mice have also played a critical role in exploring the importance of amino acids (such as arginine, tryptophan, and glutamine) in the biology of tumor-infiltrating lymphocytes (TILs). The scarcity of these amino acids in the TME can drive dysfunction of CAR-T cells and TILs. The essential role of arginine in T-cell activation, proliferation, and cytokine production has been demonstrated in TCR transgenic mouse models of CD4+ T cells specific for the influenza HA110–119 peptide (135). Sustained Arg1 activity require transport of arginine into the cell through cationic amino acid transporters. CAT2B is thought to be the most efficient in the transport of arginine into cells. Indeed, M-MDSCs or PMN-MDSCs derived from Cat2^-/-^ mice adoptively transferred in tumor-bearing mice displayed a significantly reduced ability to inhibit T cell proliferation in *in vitro* assays (136). This was confirmed *in vivo* in a thymoma tumor model, where the anti-tumor activity of adoptively transferred antigen-specific CD8+ T cells was significantly enhanced in Cat2^-/-^ *vs*. wild-type mice as a result of decreased ability of Cat2^-/-^ MDSCs to suppress T cell function and proliferation (136, 137).

#### 4.1.2 Soluble factors

In addition to its role in forcing metabolic reprogramming, hypoxia induces the accumulation of extracellular adenosine. The ectonucleotidases CD39 and CD73 lead to the sequential dephosphorylation of extracellular ATP and their expression on tumour cells, immune cells, fibroblasts, endothelial cells, and stromal cells is upregulated by hypoxia and TGFβ in the TME (138). Activated T effector cells can sense extracellular adenosine through the adenosine A2A receptor (A2AR), which triggers accumulation of intracellular cyclic adenosine monophosphate (cAMP) and limits TCR-mediated activation and expansion of effector T cells (139).

Numerous preclinical studies have demonstrated the potential advantages of inhibiting the adenosine pathway to augment the *in vivo* function of CAR-T cells (140–142). However, it is worth noting that the regulation of the adenosine pathway differs significantly between mice versus human. In mice, Tregs express CD39 and exhibit high levels of CD73 on their cell surface. This allows them to degrade ATP and generate adenosine, which has a dual impact: inhibiting effector T cells while enhancing the suppressive capabilities of Tregs (143, 144). Conversely, in the human T cell compartment, CD73 expression is primarily observed on the surface of naïve CD8 T cells and is only present in a small fraction of mature CD4 and CD8 memory T cells (145). Importantly, CD73 expression on Tregs is nearly absent, making the co-expression of CD73 with CD39 on Tregs a rare occurrence (146).

Transforming growth factor-β (TGFβ) is produced by multiple cells in the TME, and its expression is further enhanced by hypoxia (147). Due to its potent immunosuppressive effect and widespread presence in solid tumors, TGFβ has emerged as a popular target for cell therapy. Two commonly used strategies to modulate TGFβ activity involve the expression of dominant negative TGFβRII or targeted knockout (KO) of TGFβRII (148–155). The complex nature of TGFβ poses challenges for systemic therapies, as they often result in a wide range of side effects. In contrast, intrinsic T cell approaches offer a more targeted solution that avoids these issues. However, it’s important to note that testing the impact of TGFβ inhibition in cell therapy often relies on xenograft mouse models. While these models provide valuable insights, they do not fully replicate certain aspects such as lymphopenic proliferation following LDC or the interaction with endogenous adaptive immune cells, which are known to be influenced by TGFβ (156, 157). Therefore, the use of syngeneic or humanized murine models may be crucial to fully elucidate the effects of TGFβ-based armoring strategies on cell therapies.

#### 4.1.3 Inhibitory molecules

Engineering inhibitory molecules to bypass negative signaling is a promising approach to enhance CAR-T cell accumulation. Inhibition of the programmed death-1 (PD-1) checkpoint pathway through the expression of a dominant negative PD-1 receptor (PD-1 DNR) has shown efficacy in enhancing the antitumor function of mesothelin-targeted CAR-T cells in xenograft models of pleural mesothelioma with high PD-1 expression (158). PD-1 DNR expression demonstrated superior efficacy compared to systemic PD-1 blockade, which required multiple doses to achieve effectiveness. Additionally, fusion receptors combining the PD-1 ectodomain and CD28 endodomain, known as switch receptors, have also been shown to enhance the antitumor function of CAR-T cells in xenograft models (158–160).

In addition to PD-1, CAR-T cells have been equipped with a dominant negative receptor for tumor necrosis factor receptor superfamily member 6 (TNFRSF6), also known as FAS. In syngeneic tumor models, expression of FAS DNR in CAR- or TCR-engineered T cells protected the cells from FAS ligand (FASL)-induced apoptosis, leading to improved *in vivo* persistence and tumor eradication (161). Lymphocyte activation gene-3 (LAG-3) knockout CAR-T cells have also been tested in xenograft models (162). However, when exploring inhibitory molecules like CTLA-4, which requires interaction with its ligands CD80 and CD86 expressed on myeloid cells, the use of syngeneic or humanized models becomes crucial (163).

### 4.2 Accumulation - offensive strategies

#### 4.2.1 Cytokines

Improving the effectiveness of CAR T-cell therapy hinges on strategies that can shape the immune regulatory milieu within tumor tissue. In this context, offensive armoring strategies play a significant role. One approach is to engineer CAR-T cells with a transgenic cytokine that can overcome the insufficient production of pro-inflammatory factors in the TME. The transgenic cytokine can provide autocrine stimulation to sustain the survival and amplification of CAR-T cells, as well as paracrine modulation of the endogenous immune cell environment, thereby reducing systemic toxicity and remodeling the TME.

IL-12 has been studied as a cytokine for armoring CAR T cells, and its antitumor efficacy has been demonstrated in xenograft and immunocompetent syngeneic mouse models of ovarian cancer or hepatocellular carcinoma (164, 165). To further enhance the therapeutic potential of IL-12-secreting CAR T cells, Zhang et al. developed an NFAT-inducible membrane-bound IL-12, combining tumor-specific and inducible T-cell-mediated delivery with membrane-restricted localization (166). However, establishing a direct correlation between preclinical data and clinical toxicity remains challenging, as toxicity associated with IL-12 utilization is a primary concern. Variability in immune responses and toxicities, which occur in a dose-dependent manner, must be carefully considered in ongoing and future clinical trials (167).

IL-18 is another proinflammatory cytokine being explored in preclinical models and early clinical trials for cell therapy. Similar to IL-12, the efficacy of CAR T cells engineered to secrete IL-18 has been studied in xenograft and syngeneic mouse models (168, 169). Murine T cells expressing 19m28mz-P2A-mIL18 CAR were infused in C57BL/6 hCD19+/- mCD19+/- mouse model (169). This immune-competent, conditioning-dependent, tumor model uses systemic hCD19 modified EL4 (EL4[hCD19]) thymoma tumors infused into C57BL6 mice with a knockout murine CD19 (mCD19−/−), knock-in human CD19 (hCD19+/−) phenotype (C57BL6 [mCD19−/− hCD19+/−]) (170). These mice have restricted expression of one functional copy of the hCD19 gene in normal B cells, resulting in a retained immune-competent phenotype (170, 171). This model showed that IL-18 secreting CAR-T cells are efficacious in the absence of lymphodepleting chemotherapy. IL-18 secreting CD19 CAR-T demonstrated encouraging efficacy in an early clinical trials or the treatment of patients with relapsed/refractory B-cell non-Hodgkin lymphomas (NHL) or chronic lymphocytic leukemia (CLL) (NCT04684563) (172).

IL-15 is a pleiotropic cytokine that plays a role in innate and adaptive immune cell homeostasis and peripheral immune function. CD19 CAR T cells, IL-13Rα2 CAR T cells, and GD2 CAR T cells engineered to secrete IL-15 have shown increased expansion and *in vivo* anti-tumor activity in xenograft mouse models of Burkitt lymphoma, glioma, and metastatic neuroblastoma, respectively (173–175). However, there are concerns about prolonged exposure to IL-15, as IL-15 transgenic mice developed leukemia with a T-cell or NK-cell phenotype (176). The use of IL-15 to enhance the performance of CAR T cells will be further discussed in the section on longevity.

#### 4.2.2 Direct targeting of immunosuppressive populations

Enhancing the accumulation of GML in the TME can be achieved by directly targeting and eliminating immunosuppressive cell populations, as discussed earlier for CAFs and MDSCs. Tumor-associated macrophages (TAMs) are another important target in cancer treatment due to their abundance in tumors and association with poor prognosis (177). One example of targeting TAMs is the use of CAR-T cells directed against CD123, which is expressed on both Hodgkin lymphoma (HL) cells and TAMs within the TME. These CAR-T cells recognize and eliminate both HL cells and TAMs, overcoming immunosuppression and exhibiting sustained clearance in xenograft models of progressive Hodgkin lymphoma. In mouse models, rechallenging with the same tumor cell line resulted in controlled tumor growth, suggesting a protective memory response (178). However, caution should be exercised when interpreting long-term survival and persistence of T cells in immunocompromised mice, considering the role of homeostatic cytokines, potential compensatory mechanisms, and the broader context of human immunology.

Another approach involves equipping CAR-T cells with an additional CAR targeting FRβ, which is specifically expressed on immunosuppressive TAMs (114). Specific targeting and elimination of TAMs was addressed in the previous section as a means of improving infiltration into solid tumors, however, this strategy can have benefits in repolarizing the suppressive TME. In syngeneic murine tumour models of C57BL/6 mice bearing ID8 RFP-fLuc tumors (which lack mFRβ expression), transfer of CAR-T cells following cyclophosphamide preconditioning selectively eliminated FRβ+ TAMs, recruited pro-inflammatory monocytes and tumor-specific CD8+ T cells, and reduced tumor growth. This targeting approach shows promise in modulating TAMs and enhancing the antitumor immune response.

Regulatory T cells (Tregs) can also be targeted by cellular immunotherapies. However, systemic blockade of Tregs has led to life-threatening autoimmune side effects in patients due to depletion of CD25+ effector T cells. While there is early *in vitro* proof-of-concept showing that NK-92 cells expressing an anti-CD25 CAR can target Tregs, *in vivo* data are lacking, and concerns about on-target, off-tumor toxicity remain (179). *In vivo* studies in syngeneic models have shown that an immunotoxin targeting CD25 can selectively kill intratumoral Tregs while boosting endogenous immunity (180). Although incorporating such an approach into GML-based therapy is tempting, further studies are required to better understand the risk of on-target, off-tumor toxicities.

## 5 Longevity

**Figure 5:**
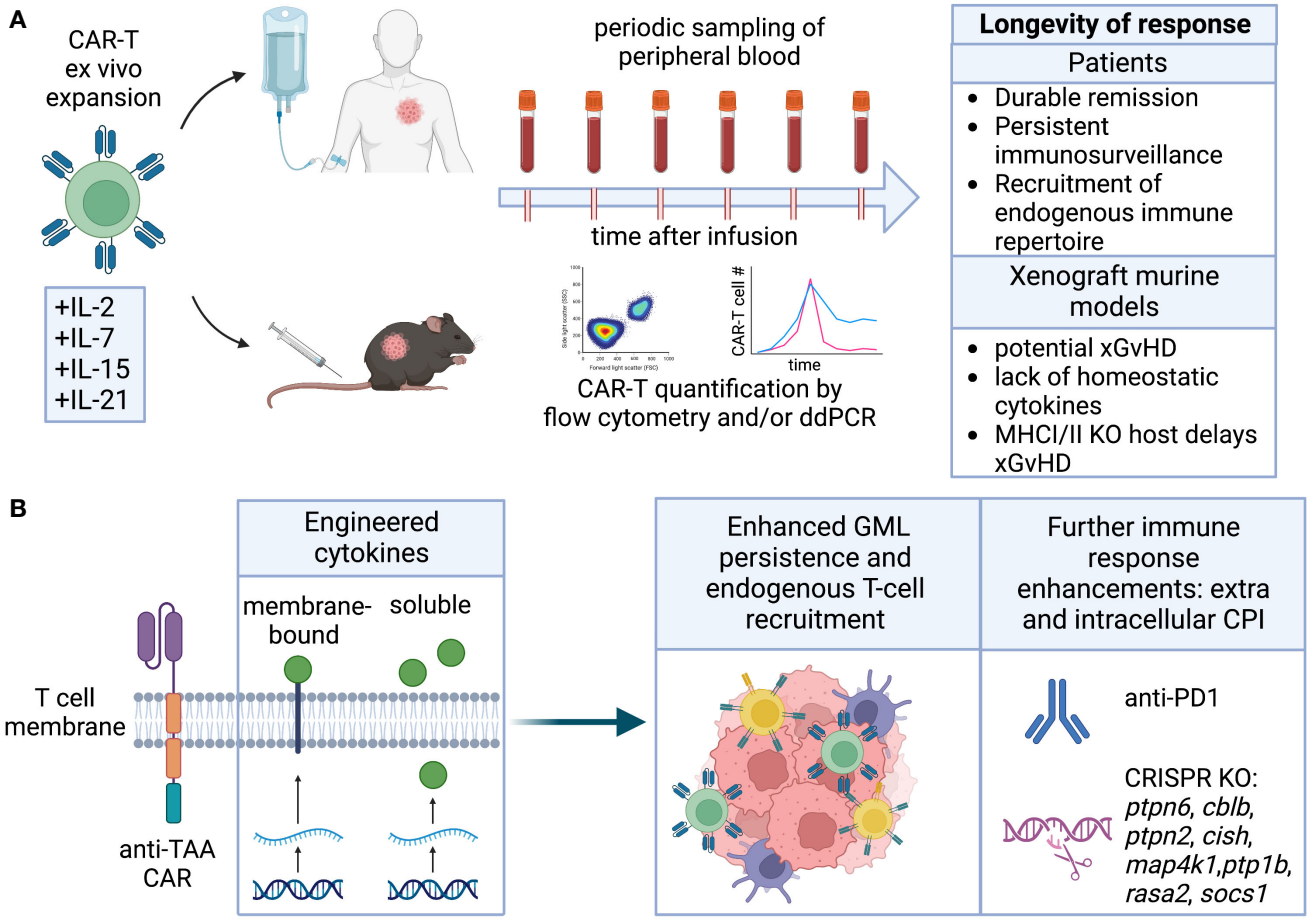
Longevity. **(A)** Cytokines employed for CAR-T ex vivo expansion can affect their differentiation and ability to self-renew and persist in the host upon infusion. Longevity is measured by quantification of CAR-T in peripheral blood, and results in durable remission. Persistence of human CAR-T in immunocompromised mice is complicated by potential GvHD and lack of homeostatic cytokines. **(B)** Engineering of cytokines enhancing CAR-T cell stemness and fitness directly or indirectly improves endogenous T cell recruitment. CRISPR KO of intracellular checkpoint inhibitors or combination with CPI blocking antibodies can further improve CAR-T persistence.

One of the unique characteristics of GML which sets it apart from traditional therapeutics, is its ability to persist in the host for months or even years. This prolonged persistence enables GML to potentially drive long-term disease remission and provide ongoing immunosurveillance against residual tumor cells (**Figure 5A**). The potential for long-term persistence is becoming increasingly evident over time, as seen in patients with leukemia who have experienced 10 or more years of clinical remission with ongoing evidence of CD19 CAR-T cell function (181). While studies have demonstrated long-term engraftment of memory CD8 cells in mice through serial transfer (182), there is currently no known research on the persistent functional exposure of GML in mice.

Long-term engraftment of human T cells in NOD-scid IL2Rγnull (NSG) mice is complicated by xenograft-versus-host (xGvHD) immune-reactivity. This immune reactivity arises from both the MHC mismatch between human and mouse and the compromised immune system of the NSG mice, which is unable to reject the human T cell graft. Consequently, long-term engraftment studies in NSG mice may be confounded by toxicity that is specific to the model and may not accurately represent autologous GML therapy in humans. While NSG mice with Class I and Class II MHC knock-out can attenuate this toxicity (183, 184), they remain severely immunocompromised and may still generate xGvHD responses through the interaction of non-classical MHC molecules (e.g., CD1) or stress molecules (MICA/B) (185). Therefore, conducting short-term studies (~30 days) or using mouse models that can predict the consequences of long-term engraftment and endogenous immune conditioning and repertoire spreading may be more feasible and informative. Immunocompetent or syngeneic models, on the other hand, are not often used to study persistence due to challenges with T cell engineering, expansion, and the lack of models with slow tumor growth and endogenous antigen expression. Nonetheless, there are examples suggesting that transduced and expanded murine GML can be effective and persistent, and further development of these models is warranted (186).

Modifications aimed at enhancing GML longevity encompass various approaches, including alterations to intracellular domains, introduction of cytokines through genetic or pharmacological means, and deletion of intracellular inhibitory proteins that limit lymphocyte function, cytotoxicity, and persistence. These strategies not only promote the longevity of GML but can also be utilized to recruit endogenous lymphocytes, thereby extending the durability of the therapeutic response (**Figure 5B**).

### 5.1 Longevity – co-stimulatory domains

Early clinical trials with CD19 CAR T cells demonstrated that incorporating the 4-1BB co-stimulatory domain in the intracellular domain (ICD) of second-generation CARs, referred to as BBζ, resulted in enhanced persistence compared to CARs containing the CD28 co-stimulatory domain (CD28ζ). The enhanced persistence of BBζ CAR-T cells was attributed to 4-1BB’s ability to activate TRAF/NF-κB signaling, promoting fatty acid oxidation and mitochondrial biogenesis, which contrasted with the predominantly glycolytic signaling downstream of CD28 (187). Clinical data showed that CD28ζ CAR-T persistence rarely exceeded 42 days (188, 189), while BBζ CAR-T persistence often exceeded 1 year in patients achieving complete responses (190).

Subsequent investigations consistently revealed distinct metabolic responses between CD28ζ and BBζ CAR-T cells, with CD28ζ CAR-T cells exhibiting a “fast burn” metabolic response driven by PI3K-induced aerobic glycolysis, and BBζ CAR-T cells displaying a “slow burn” oxidative metabolism primarily fueled by fatty acid breakdown, aligning with 4-1BB’s role in T memory development and longevity (191). These findings indicated that the design of the CAR ICD can directly influence its persistence. Researchers have since developed various versions of second-generation CARs containing additional costimulatory domains such as ICOS and OX40 (192, 193), as well as third-generation CARs, aiming to enhance the balance between anti-tumor cytotoxicity and persistence (194, 195). While these versions have demonstrated strong activity in mouse xenograft tumor models using NSG mice, clinical evidence of persistence superior to second-generation BBζ CAR-T cells in humans is yet to be established (196).

Although preclinical mouse models cannot fully replicate the persistence of engrafted T cells observed in humans, studies comparing BBζ and CD28ζ CAR-T cell performance in standard xenograft models have shown similar tumor-clearing effects between the two at high doses. However, at lower doses, BBζ CAR-T cells demonstrated functional superiority, indicating better memory conversion and resistance to the stress of low cell dosing (197). While mouse models can indirectly predict the longevity and persistence of GML based on the design of the CAR’s intracellular domain (ICD), it is essential to acknowledge the limitations of these models in fully replicating the long-term engraftment and endogenous immune-conditioning observed in humans.

### 5.2 Longevity – role of cytokines

In clinical trials for TIL and TCR-T therapy, IL-2 is commonly used to support the function and persistence of the adoptively transferred cells. However, in the case of CAR-T therapy, IL-2 is rarely used to enhance CAR-T persistence. This may be attributed, in part, to the CAR-T cells’ ability to produce paracrine IL-2 through the engagement of co-stimulatory CAR intracellular domains (ICD). Additionally, IL-2 is avoided in GML therapy due to the toxicities associated with higher doses needed to maintain the anti-tumor function of transferred T cells (198).

Recent advancements have led to the development of orthogonal versions of IL-2 that can be genetically engineered into CAR-T cells, promoting expansion and longevity with limited systemic toxicities (199, 200). By engineering CD19 CAR-T cells to express mutated IL-2Rβ receptors, which can only be activated by cell-encoded, mutated orthogonal IL-2 (ortho-IL-2), selectivity can be achieved without encountering the negative effects of IL-2 binding on wild-type T or NK cells. Two separate groups have described this mutant IL-2 “orthokine” pair and demonstrated its effectiveness in combination with suboptimal doses of CAR-T cells, leading to CAR-T expansion, tumor reduction, and persistence without the need for repeated CAR-T infusions. Interestingly, in preclinical xenograft models using NSG mice, higher doses of ortho-IL-2 were found to induce weight loss, although this toxicity was determined to be unrelated to xGvHD based on studies with TCR knockout mice. The authors speculated that the observed toxicity, which occurred at doses much higher than those required for tumor regression, was target-dependent, as weight loss and toxicity were observed in mice bearing NALM6 tumors but not in tumor-deficient mice (201). A human clinical trial testing the IL-2 orthokine and CAR-T pairing (STK-009) is currently enrolling patients with relapsed or refractory CD19 positive hematologic malignancies to evaluate the combination of autologous CD19 CAR-T cells with orthogonal IL-2 (201). This trial aims to assess the safety and efficacy of this approach in a clinical setting.

The differentiation status of T cells directly influences their longevity and antitumor activity. T cells with a stem-like phenotype, characterized by enhanced self-renewal and proliferation capacity, are associated with better clinical responses (202–207). Manipulating the differentiation status of T cells is a commonly explored strategy to improve the quality and efficacy of GML. Common-gamma chain cytokines such as IL-7 and IL-15 are frequently used in cell therapy to either promote a less differentiated product during the expansion stage or engineered into GML as an armoring strategy for *in vivo* enhancement (207).

IL-15 plays a crucial role in the homeostasis and development of CD8+ T cells by inhibiting activation-induced cell death (AICD) and upregulating antiapoptotic mediators like Mcl1 and Bcl-2 (208). IL-15-secreting CAR-T cells exhibited a less differentiated phenotype compared to unarmored CAR-T cells in a syngeneic model of subcutaneous B16 melanoma. They also showed reduced expression of PD1, prolonged persistence, and superior cellular activity (48). In another study, IL-15-armored CAR-T cells completely prevented tumor relapse in a xenograft model but were associated with severe liver toxicity and a GvHD score (209). Clinical studies have also reported IL-15 administration-related toxicity in cancer patients (210).

To mitigate IL-15 toxicity, researchers generated CD19 CAR-T cells that overexpressed both IL-15 and IL-15 receptor alpha. This strategy successfully blocked toxicity while maintaining the persistence and anti-tumor activity of CAR-T cells induced by IL-15 (209). However, the observed toxicity in mice is a subject of debate, as another study found that CD19 CAR-T cells expressing membrane-bound chimeric IL-15 delayed leukemia development, exhibited sustained resistance after tumor clearance, and generated long-lived TSCM cells without any associated toxicity (211).

Interleukin-7 (IL-7) plays a crucial role in enhancing the homeostasis and survival of memory and naïve T cells by upregulating Bcl-2 and repressing proapoptotic markers (212). Several studies have demonstrated that *in vitro* expansion of CAR-T cells with IL-7 leads to a higher frequency of cells exhibiting a memory stem cell phenotype (33). These cells retained a less differentiated phenotype and exhibited increased resistance to dysfunction upon repetitive encounters with antigens.

Early reports showed that constitutive expression of IL-7 enhanced the *in vivo* efficacy of CD19 CAR-T cells in a xenograft model of leukemia. However, it did not improve long-term persistence compared to CAR-T cells expressing IL-2 or IL-15 (213). IL-7-expressing CLDN18.2 CAR-T cells demonstrated increased proliferation, decreased apoptosis, and lower expression of exhaustion markers compared to unarmored CAR-T cells (214). However, IL-7 signaling specifically suppresses IL7Rα transcription as a homeostatic regulatory mechanism, promoting the survival of the maximum possible number of T cells given the available IL-7 (215).

To overcome the potential loss of IL-7 responsiveness, IL-7Rα was engineered into EBV-CTLs, resulting in an efficient response to IL-7 and *in vivo* activity in a xenograft model (216). Similarly, the introduction of a constitutively active IL-7 receptor (C7R) improved the *in vivo* antitumor activity of GD2-CAR-T cells in a xenograft model of metastatic neuroblastoma and EphA2-CAR-T cells in a orthotopic xenograft model of glioblastoma (217). It is important to note that GD2-CAR-T cells expressing C7R are currently being tested against relapsed or refractory neuroblastoma and other GD2-positive cancers (NCT03635632). Although autonomous or antigen-independent proliferation was not reported in this study, activating mutations of IL7R are present in 10% of pre-T-cell acute leukemias, raising concerns about unwanted proliferation associated with this approach.

Interleukin-21 (IL-21) is a crucial cytokine involved in regulating the function of CD8+ T cells and has emerged as a potential target for cancer immunotherapy. IL-21 enhances CD8+ T cell activity by promoting their proliferation, differentiation, and increasing the expression of cytotoxic molecules like perforin and granzyme B (218). Additionally, IL-21 indirectly enhances anti-tumor activity by promoting antibody production by B cells, aiding in targeting and killing tumor cells (219).

Recent studies have demonstrated the effectiveness of IL-21 in improving the anti-tumor effect of CAR-T cells. GPC3 CAR-T cells expressing IL-15 and IL-21 exhibited significantly greater anti-tumor efficacy in mice compared to CAR-T cells without IL-21 expression (220). These IL-21 expressing CAR-T cells are currently being evaluated in clinical trials for the treatment of hepatocellular carcinoma (HCC) (NCT02932956 and NCT02905188). Another study utilized NFAT-inducible secreted IL-21, which demonstrated increased anti-tumor efficacy in NSG mice and resistance to immune suppression induced by chronic lymphocytic leukemia cells (221). Engineered switch receptors have also been explored to provide IL-21 signaling. Wang et al. tested a receptor composed of the IL-4 ectodomain and the IL-21 endodomain and compared it to a receptor with the IL-7 endodomain (222). The IL-4/IL-21 inverted receptor conferred enhanced *in vivo* efficacy to GPC3 CAR-T cells against a xenograft model expressing varying levels of GPC3, compared to unarmored CAR-T cells or those with the IL-4/IL-7 inverted receptor. The study also observed a higher number of CD3+ T cells in the peripheral blood of mice receiving cells expressing the IL-4/IL-21 inverted receptor, indicating improved *in vivo* persistence (222). However, as previously mentioned, conclusions about *in vivo* persistence in xenograft models should be interpreted with caution.

Another interesting approach is the incorporation of a motif for STAT3 recruitment at the C-terminus of CD3ζ, enabling IL-21 signaling upon CAR engagement (223). CD19 CAR-T cells expressing this chimeric receptor demonstrated enhanced proliferation and cytokine polyfunctionality compared to control CAR-T cells in xenograft models of NALM6 human leukemia cells and subcutaneous CD19+ tumors, suggesting a key role for STAT3 in suppressing terminal differentiation of T cells (223–225).

### 5.3 Longevity – blocking inhibitory mechanisms

GML therapy can benefit from strategies that overcome co-inhibitory mechanisms, which naturally restrict long-term proliferation of T cells. One approach is to induce intra-tumoral production of checkpoint inhibitor (CPI) antibodies, antibody-like fragments, or soluble PD1. Several studies have reported the use of CAR-T cells engineered to secrete constitutive or inducible CPI in mouse models (226–228). While these preclinical studies in xenograft mouse models have shown improved CAR-T function, such as enhanced expansion, cytolytic activity, or effector function, similar enhancements have not yet been observed in human clinical trials (229, 230). It is worth noting that the human trials utilized CPI in combination with CD19 CAR-T for CPI-insensitive indications, leaving open the possibility of using CPI-secreting CAR-T for CPI-sensitive tumors. However, since multiple versions of CPI antibodies are available as co-therapy, it remains unclear whether there are advantages or disadvantages to driving their expression from the GML itself. Further research is needed to elucidate the potential benefits and drawbacks of this approach.

Additional T cell checkpoints can hinder the expansion of GML, although their impact on longevity remains uncertain. Knocking down CTLA-4 expression using shRNA did not enhance the function or persistence of CD19 CAR-T cells based on 28ζ, but co-expression of CD80 alongside a first-generation CAR containing CD3ζ significantly increased expansion when CTLA-4 was knocked down (231). Currently, there are no known human clinical trials combining the CTLA-4 antibodies ipilumumab or tremelimumab with CAR-T or TCR-T. Trials testing CAR-T cells that produce CTLA-4 and PD1 antibodies against MUC1, EGFR, and MSLN are enrolling, but no preclinical or clinical data have been reported (NCT03179007, NCT03182816, NCT03182803). Blockade of LAG3 using CRISPR/Cas-mediated knockout did not show benefit in *in vitro* preclinical models but was not tested in mice (162). On the other hand, blockade of PD1 and Tim3, either alone or in combination, improved the activity of CD123-directed CAR-T cells in xenograft models of AML (232). Other T cell checkpoints include soluble molecules such as TGFβ and adenosine, which, as discussed in the previous section, can regulate both GML accumulation and longevity. Moreover, intracellular T cell checkpoints have been validated through gene deletion or expression of dominant-negative receptors (DNR), revealing additional mechanisms that can limit GML expansion. Several molecules, including SHP-1, CBLB, PTPN2, CISH, HPK1, PTP1B, RASA2, and SOCS1, have been identified as intrinsic checkpoints that can constrain T cell effector function, accumulation, and expansion (233–240). While targeting these intrinsic checkpoints individually is an active area of research, combinations of checkpoint deletions have also emerged (241). For instance, double knockout (DKO) of both regnase-1 and roquin-1 greatly increased T cell effector function, accumulation, and expansion in xenograft models compared to single knockout alone. However, the DKO also resulted in uncontrolled lymphoproliferation and toxicity in some mice. This finding emphasizes the need to understand the interaction of intracellular checkpoints in GML and develop mechanisms to prevent their uncontrolled expansion in patients, as unregulated persistence can have undesirable consequences.

## 6 Discussion

“All models are wrong, but some are useful” – George E.P. Box.

GML have transformed the therapeutic landscape for multiple B cell malignancies and are making steady progress into additional tumor types and even autoimmune diseases. As with any evolving therapeutic class, GML are undergoing continual modifications to improve their activity and safety profiles in order to broaden their applicability. As the diseases that can be targeted with GML are theoretically unlimited, the solution set for therapeutic design will inevitably diverge from those used for the treatment of B cell malignancies. In order for these designs to be validated and implemented, preclinical models are required.

Until robust three-dimensional or organotypic models that can recapitulate human tumor and normal tissue biology are readily available, GML development will continue to be dependent on the mouse as the default preclinical model to understand safety and efficacy profiles before human clinical trials. Mouse models for GML are simultaneously useful and distracting. They can be very useful to understand on-target activity and efficacy, acute GML expansion and pharmacodynamics, and proximal effector functions such as cytokine release. However, they are distracting in that they miss key aspects of GML challenges such as off-tumor toxicities, representative secondary cytokine secretion from myeloid, epithelial, or stromal cells, and control of metastatic and heterogenous disease similar to human tumors. Additional artifacts include xenograft-versus-host reactivity of human T cells engrafted in NSG mice, lack of validation of fully murinized GML designs and protocols, differences in lymphodepletion regimens, and studies in pathogen-free facilities that do not account for the influence of natural indigenous gut microbiota.

Due to the varied and unpredictable behavior of GML between mice and humans, a framework called “DIAL” has been developed to compartmentalize and study individual GML behaviors. The framework allows for understanding of aspects of the cell journey that can studied and optimized in isolation. For example, distribution studies which require vaccine-like cell priming can be studied using immunocompetent syngeneic mouse models while human GML manufacturing optimization may be best studied in NSG mice. Infiltration can be explored using genetically engineered mouse models that mimic tumor fibrosis (242), and early passage patient-derived xenograft (PDX) models in NOG or NSG mice might retain sufficient stroma to prevent immune-infiltration and exclude unmodified GML (243). PDX models can likewise be used to study accumulation, where native human tumor architecture, soluble factors, and hypoxia can affect GML survival. Likewise, syngeneic models can be deployed to complement the PDX model to capture immunosuppressive facets of TME that prevent accumulation. Strategies to enhance GML longevity can be limited due to technical factors such as development of xGvHD (NSG mice) and the challenges of optimizing murine GML manufacturing or treating rapidly growing tumors in most syngeneic models. Still, certain cytokines and intracellular designs have the potential to improve GML activity in NSG mice at suboptimal doses, which can serve as surrogates for extended longevity. Longevity of GML response can also be demonstrated using tumor rechallenge studies in immunocompetent mice, demonstrating that GML-induced long-term immunity can be acquired via the expansion of endogenous anti-tumor T cells (244). These studies provide rationale for modification of GML with membrane bound cytokines or checkpoint inhibitors to bring about long-lasting remissions against heterogeneous tumors.

Following the success of CAR and TCR-T therapies in multiple tumor types, next-generation approaches will incorporate increasing complexity through genetic editing and engineering to augment GML functions. As each of these functions require validation through mechanistic and safety data, extensive preclinical testing in animals will be highly warranted if not required. While mice remain the most popular animal model for preclinical GML testing, canine and non-human primate (NHP) studies have also been conducted. Studies in non-murine species facilitate the study of unique GML attributes against tumors; canines present with spontaneous and heterogeneous tumors and are pathogen-exposed, as are several NHP species that provide the most genetically similar model to human, and present the most relevant model to recapitulate the consequences of LDC as well as allowing longitudinal sampling (245). Until non-murine models are more consistently used and understood, however, a mixture of murine models to deconstruct multiple GML functions will still be preferred. Future development of complex GML may benefit from co-evolution of efficient and specific murine and human gene-editing tools and vectors to more accurately predict these functions preclinically in order to decrease drug development cycle times, and improve efficacy and safety of the next wave of cell-based therapeutics.

## Author contributions

GM: Conceptualization, Supervision, Writing – original draft, Writing – review & editing. MT: Writing – original draft, Writing – review & editing.

## References

[1] Miller JF. Immunological function of the thymus. Lancet (1961) 2(7205):748–9. doi: 10.1016/S0140-6736(61)90693-6 14474038

[2] Cerottini JC Nordin AA Brunner KT. *In vitro* cytotoxic activity of thymus cells sensitized to alloantigens. Nature (1970) 227(5253):72–3. doi: 10.1038/227072a0 4987218

[3] Miller JF Sprent J. Cell-to-cell interaction in the immune response. VI. Contribution of thymus-derived cells and antibody-forming cell precursors to immunological memory. J Exp Med (1971) 134(1):66–82. doi: 10.1084/jem.134.1.66 5105057 PMC2139027

[4] Rosenberg SA Aebersold P Cornetta K Kasid A Morgan RA Moen R. Gene transfer into humans–immunotherapy of patients with advanced melanoma, using tumor-infiltrating lymphocytes modified by retroviral gene transduction. N Engl J Med (1990) 323(9):570–8. doi: 10.1056/NEJM199008303230904 2381442

[5] Rosenberg SA Spiess P Lafreniere R. A new approach to the adoptive immunotherapy of cancer with tumor-infiltrating lymphocytes. Science (1986) 233(4770):1318–21. doi: 10.1126/science.3489291 3489291

[6] Gross G Waks T Eshhar Z. Expression of immunoglobulin-T-cell receptor chimeric molecules as functional receptors with antibody-type specificity. Proc Natl Acad Sci U S A (1989) 86(24):10024–8. doi: 10.1073/pnas.86.24.10024 PMC298636 2513569

[7] Maher J Brentjens RJ Gunset G Riviere I Sadelain M. Human T-lymphocyte cytotoxicity and proliferation directed by a single chimeric TCRzeta /CD28 receptor. Nat Biotechnol (2002) 20(1):70–5. doi: 10.1038/nbt0102-70 11753365

[8] Imai C Mihara K Andreansky M Nicholson IC Pui CH Geiger TL. Chimeric receptors with 4-1BB signaling capacity provoke potent cytotoxicity against acute lymphoblastic leukemia. Leukemia (2004) 18(4):676–84. doi: 10.1038/sj.leu.2403302 14961035

[9] Gong MC Latouche JB Krause A Heston WD Bander NH Sadelain M. Cancer patient T cells genetically targeted to prostate-specific membrane antigen specifically lyse prostate cancer cells and release cytokines in response to prostate-specific membrane antigen. Neoplasia (1999) 1(2):123–7. doi: 10.1038/sj.neo.7900018 PMC1508130 10933046

[10] Brentjens RJ Latouche JB Santos E Marti F Gong MC Lyddane C. Eradication of systemic B-cell tumors by genetically targeted human T lymphocytes co-stimulated by CD80 and interleukin-15. Nat Med (2003) 9(3):279–86. doi: 10.1038/nm827 12579196

[11] van der Stegen SJ Hamieh M Sadelain M. The pharmacology of second-generation chimeric antigen receptors. Nat Rev Drug Discovery (2015) 14(7):499–509. doi: 10.1038/nrd4597 26129802 PMC6410718

[12] Kochenderfer JN Wilson WH Janik JE Dudley ME Stetler-Stevenson M Feldman SA. Eradication of B-lineage cells and regression of lymphoma in a patient treated with autologous T cells genetically engineered to recognize CD19. Blood (2010) 116(20):4099–102. doi: 10.1182/blood-2010-04-281931 PMC2993617 20668228

[13] Johnson LA Heemskerk B Powell DJ Jr. Cohen CJ Morgan RA Dudley ME. Gene transfer of tumor-reactive TCR confers both high avidity and tumor reactivity to nonreactive peripheral blood mononuclear cells and tumor-infiltrating lymphocytes. J Immunol (2006) 177(9):6548–59. doi: 10.4049/jimmunol.177.9.6548 PMC2174608 17056587

[14] Overwijk WW Theoret MR Finkelstein SE Surman DR de Jong LA Vyth-Dreese FA. Tumor regression and autoimmunity after reversal of a functionally tolerant state of self-reactive CD8+ T cells. J Exp Med (2003) 198(4):569–80. doi: 10.1084/jem.20030590 PMC2194177 12925674

[15] Harris JE Harris TH Weninger W Wherry EJ Hunter CA Turka LA. A mouse model of vitiligo with focused epidermal depigmentation requires IFN-gamma for autoreactive CD8(+) T-cell accumulation in the skin. J Invest Dermatol (2012) 132(7):1869–76. doi: 10.1038/jid.2011.463 PMC3343174 22297636

[16] Dudley ME Wunderlich JR Robbins PF Yang JC Hwu P Schwartzentruber DJ. Cancer regression and autoimmunity in patients after clonal repopulation with antitumor lymphocytes. Science (2002) 298(5594):850–4. doi: 10.1126/science.1076514 PMC1764179 12242449

[17] Research CfBEa Administration FaD. Considerations for the development of chimeric antigen receptor (CAR) T cell products. (2022). Available at: https://www.fda.gov/regulatory-information/search-fda-guidance-documents/considerations-development-chimeric-antigen-receptor-car-t-cell-products.

[18] Zhu H Melder RJ Baxter LT Jain RK. Physiologically based kinetic model of effector cell biodistribution in mammals: implications for adoptive immunotherapy. Cancer Res (1996) 56(16):3771–81. 8706023

[19] Mackay CR Marston WL Dudler L. Naive and memory T cells show distinct pathways of lymphocyte recirculation. J Exp Med (1990) 171(3):801–17. doi: 10.1084/jem.171.3.801 PMC2187792 2307933

[20] Koya RC Mok S Comin-Anduix B Chodon T Radu CG Nishimura MI. Kinetic phases of distribution and tumor targeting by T cell receptor engineered lymphocytes inducing robust antitumor responses. Proc Natl Acad Sci U S A (2010) 107(32):14286–91. doi: 10.1073/pnas.1008300107 PMC2922609 20624956

[21] Smith ME Ford WL. The recirculating lymphocyte pool of the rat: a systematic description of the migratory behaviour of recirculating lymphocytes. Immunology (1983) 49(1):83–94. 6840811 PMC1454101

[22] Liu C Ayyar VS Zheng X Chen W Zheng S Mody H. Model-based cellular kinetic analysis of chimeric antigen receptor-T cells in humans. Clin Pharmacol Ther (2021) 109(3):716–27. doi: 10.1002/cpt.2040 PMC7959329 33002189

[23] Parente-Pereira AC Burnet J Ellison D Foster J Davies DM van der Stegen S. Trafficking of CAR-engineered human T cells following regional or systemic adoptive transfer in SCID beige mice. J Clin Immunol (2011) 31(4):710–8. doi: 10.1007/s10875-011-9532-8 21505816

[24] Sallusto F Lenig D Forster R Lipp M Lanzavecchia A. Two subsets of memory T lymphocytes with distinct homing potentials and effector functions. Nature (1999) 401(6754):708–12. doi: 10.1038/44385 10537110

[25] Khot A Matsueda S Thomas VA Koya RC Shah DK. Measurement and quantitative characterization of whole-body pharmacokinetics of exogenously administered T cells in mice. J Pharmacol Exp Ther (2019) 368(3):503–13. doi: 10.1124/jpet.118.252858 PMC6382992 30622170

[26] Rosenberg SA Packard BS Aebersold PM Solomon D Topalian SL Toy ST. Use of tumor-infiltrating lymphocytes and interleukin-2 in the immunotherapy of patients with metastatic melanoma. A preliminary Rep N Engl J Med (1988) 319(25):1676–80. doi: 10.1056/NEJM198812223192527 3264384

[27] Rosenberg SA Yannelli JR Yang JC Topalian SL Schwartzentruber DJ Weber JS. Treatment of patients with metastatic melanoma with autologous tumor-infiltrating lymphocytes and interleukin 2. J Natl Cancer Inst (1994) 86(15):1159–66. doi: 10.1093/jnci/86.15.1159 8028037

[28] Gattinoni L Lugli E Ji Y Pos Z Paulos CM Quigley MF. A human memory T cell subset with stem cell-like properties. Nat Med (2011) 17(10):1290–7. doi: 10.1038/nm.2446 PMC3192229 21926977

[29] Sabatino M Hu J Sommariva M Gautam S Fellowes V Hocker JD. Generation of clinical-grade CD19-specific CAR-modified CD8+ memory stem cells for the treatment of human B-cell Malignancies. Blood (2016) 128(4):519–28. doi: 10.1182/blood-2015-11-683847 PMC4965906 27226436

[30] Turtle CJ Hanafi LA Berger C Gooley TA Cherian S Hudecek M. CD19 CAR-T cells of defined CD4+:CD8+ composition in adult B cell ALL patients. J Clin Invest (2016) 126(6):2123–38. doi: 10.1172/JCI85309 PMC4887159 27111235

[31] Cieri N Camisa B Cocchiarella F Forcato M Oliveira G Provasi E. IL-7 and IL-15 instruct the generation of human memory stem T cells from naive precursors. Blood (2013) 121(4):573–84. doi: 10.1182/blood-2012-05-431718 23160470

[32] Barrett DM Singh N Liu X Jiang S June CH Grupp SA. Relation of clinical culture method to T-cell memory status and efficacy in xenograft models of adoptive immunotherapy. Cytotherapy (2014) 16(5):619–30. doi: 10.1016/j.jcyt.2013.10.013 PMC3988256 24439255

[33] Xu Y Zhang M Ramos CA Durett A Liu E Dakhova O. Closely related T-memory stem cells correlate with in *vivo* expansion of CAR.CD19-T cells and are preserved by IL-7 and IL-15. Blood (2014) 123(24):3750–9. doi: 10.1182/blood-2014-01-552174 PMC4055922 24782509

[34] Gattinoni L Zhong XS Palmer DC Ji Y Hinrichs CS Yu Z. Wnt signaling arrests effector T cell differentiation and generates CD8+ memory stem cells. Nat Med (2009) 15(7):808–13. doi: 10.1038/nm.1982 PMC2707501 19525962

[35] Zheng W O'Hear CE Alli R Basham JH Abdelsamed HA Palmer LE. PI3K orchestration of the in *vivo* persistence of chimeric antigen receptor-modified T cells. Leukemia (2018) 32(5):1157–67. doi: 10.1038/s41375-017-0008-6 PMC5943191 29479065

[36] Klebanoff CA Crompton JG Leonardi AJ Yamamoto TN Chandran SS Eil RL. Inhibition of AKT signaling uncouples T cell differentiation from expansion for receptor-engineered adoptive immunotherapy. JCI Insight (2017) 2(23):e95103. doi: 10.1172/jci.insight.95103 29212954 PMC5752304

[37] Pilipow K Scamardella E Puccio S Gautam S De Paoli F Mazza EM. Antioxidant metabolism regulates CD8+ T memory stem cell formation and antitumor immunity. JCI Insight (2018) 3(18):e122299. doi: 10.1172/jci.insight.122299 30232291 PMC6237218

[38] Foskolou IP Barbieri L Vernet A Bargiela D Cunha PP Velica P. The S enantiomer of 2-hydroxyglutarate increases central memory CD8 populations and improves CAR-T therapy outcome. Blood Adv (2020) 4(18):4483–93. doi: 10.1182/bloodadvances.2020002309 PMC7509862 32941648

[39] Xia L Liu JY Zheng ZZ Chen YJ Ding JC Hu YH. BRD4 inhibition boosts the therapeutic effects of epidermal growth factor receptor-targeted chimeric antigen receptor T cells in glioblastoma. Mol Ther (2021) 29(10):3011–26. doi: 10.1016/j.ymthe.2021.05.019 PMC8531146 34058385

[40] Zhao Z Condomines M van der Stegen SJC Perna F Kloss CC Gunset G. Structural design of engineered costimulation determines tumor rejection kinetics and persistence of CAR T cells. Cancer Cell (2015) 28(4):415–28. doi: 10.1016/j.ccell.2015.09.004 PMC5003056 26461090

[41] Goff SL Morgan RA Yang JC Sherry RM Robbins PF Restifo NP. Pilot trial of adoptive transfer of chimeric antigen receptor-transduced T cells targeting EGFRvIII in patients with glioblastoma. J Immunother (2019) 42(4):126–35. doi: 10.1097/CJI.0000000000000260 PMC6691897 30882547

[42] Lugli E Dominguez MH Gattinoni L Chattopadhyay PK Bolton DL Song K. Superior T memory stem cell persistence supports long-lived T cell memory. J Clin Invest (2013) 123(2):594–9. doi: 10.1172/JCI66327 PMC3561805 23281401

[43] Cao H Kim DH Howard A Moz H Wasnik S Baylink DJ. Ex vivo isolation, expansion and bioengineering of CCR7+CD95-/or CD62L+CD45RA+ tumor infiltrating lymphocytes from acute myeloid leukemia patients' bone marrow. Neoplasia (2021) 23(12):1252–60. doi: 10.1016/j.neo.2021.11.003 PMC8603025 34775232

[44] Kishton RJ Vodnala SK Vizcardo R Restifo NP. Next generation immunotherapy: enhancing stemness of polyclonal T cells to improve anti-tumor activity. Curr Opin Immunol (2022) 74:39–45. doi: 10.1016/j.coi.2021.10.001 34710751

[45] Ghassemi S Durgin JS Nunez-Cruz S Patel J Leferovich J Pinzone M. Rapid manufacturing of non-activated potent CAR T cells. Nat BioMed Eng (2022) 6(2):118–28. doi: 10.1038/s41551-021-00842-6 PMC8860360 35190680

[46] Dickinson MJ Barba P Jager U Shah NN Blaise D Briones J. A novel autologous CAR-T therapy, YTB323, with preserved T-cell stemness shows enhanced CAR T-cell efficacy in preclinical and early clinical development. Cancer Discovery (2023) 13(9):1982–97. doi: 10.1158/2159-8290.24091927.v1 PMC10481129 37249512

[47] Lee J Sadelain M Brentjens R. Retroviral transduction of murine primary T lymphocytes. Methods Mol Biol (2009) 506:83–96. doi: 10.1007/978-1-59745-409-4_7 19110621 PMC5003426

[48] Lanitis E Rota G Kosti P Ronet C Spill A Seijo B. Optimized gene engineering of murine CAR-T cells reveals the beneficial effects of IL-15 coexpression. J Exp Med (2021) 218(2):e20192203. doi: 10.1084/jem.20192203 33156338 PMC7653685

[49] Gautam S Fioravanti J Zhu W Le Gall JB Brohawn P Lacey NE. The transcription factor c-Myb regulates CD8(+) T cell stemness and antitumor immunity. Nat Immunol (2019) 20(3):337–49. doi: 10.1038/s41590-018-0311-z PMC6489499 30778251

[50] Hanada KI Yu Z Chappell GR Park AS Restifo NP. An effective mouse model for adoptive cancer immunotherapy targeting neoantigens. JCI Insight (2019) 4(10):e124405. doi: 10.1172/jci.insight.124405 31092734 PMC6542630

[51] Vodnala SK Eil R Kishton RJ Sukumar M Yamamoto TN Ha NH. T cell stemness and dysfunction in tumors are triggered by a common mechanism. Science (2019) 363(6434):eaau0135. doi: 10.1126/science.aau0135 30923193 PMC8194369

[52] Klebanoff CA Scott CD Leonardi AJ Yamamoto TN Cruz AC Ouyang C. Memory T cell-driven differentiation of naive cells impairs adoptive immunotherapy. J Clin Invest (2016) 126(1):318–34. doi: 10.1172/JCI81217 PMC4701537 26657860

[53] Sukumar M Liu J Mehta GU Patel SJ Roychoudhuri R Crompton JG. Mitochondrial membrane potential identifies cells with enhanced stemness for cellular therapy. Cell Metab (2016) 23(1):63–76. doi: 10.1016/j.cmet.2015.11.002 26674251 PMC4747432

[54] Corria-Osorio J Carmona SJ Stefanidis E Andreatta M Ortiz-Miranda Y Muller T. Orthogonal cytokine engineering enables novel synthetic effector states escaping canonical exhaustion in tumor-rejecting CD8(+) T cells. Nat Immunol (2023) 24(5):869–83. doi: 10.1038/s41590-023-01477-2 PMC10154250 37081150

[55] Hu X Majchrzak K Liu X Wyatt MM Spooner CJ Moisan J. *In vitro* priming of adoptively transferred T cells with a RORgamma agonist confers durable memory and stemness in vivo. Cancer Res (2018) 78(14):3888–98. doi: 10.1158/0008-5472.CAN-17-3973 PMC6238208 29769201

[56] Kuan CT Wikstrand CJ Archer G Beers R Pastan I Zalutsky MR. Increased binding affinity enhances targeting of glioma xenografts by EGFRvIII-specific scFv. Int J Cancer (2000) 88(6):962–9. doi: 10.1002/1097-0215(20001215)88:6<962::AID-IJC20>3.0.CO;2-U 11093822

[57] Van Elssen CH Frings PW Bot FJ Van de Vijver KK Huls MB Meek B. Expression of aberrantly glycosylated Mucin-1 in ovarian cancer. Histopathology (2010) 57(4):597–606. doi: 10.1111/j.1365-2559.2010.03667.x 20955385

[58] Castellarin M Sands C Da T Scholler J Graham K Buza E. A rational mouse model to detect on-target, off-tumor CAR T cell toxicity. JCI Insight (2020) 5(14):e136012. doi: 10.1172/jci.insight.136012 32544101 PMC7453898

[59] Giardino Torchia ML Gilbreth R Merlino A Sult E Monks N Chesebrough J. Rational design of chimeric antigen receptor T cells against glypican 3 decouples toxicity from therapeutic efficacy. Cytotherapy (2022) 24(7):720–32. doi: 10.1016/j.jcyt.2022.03.008 35570170

[60] Richman SA Nunez-Cruz S Moghimi B Li LZ Gershenson ZT Mourelatos Z. High-affinity GD2-specific CAR T cells induce fatal encephalitis in a preclinical neuroblastoma model. Cancer Immunol Res (2018) 6(1):36–46. doi: 10.1158/2326-6066.CIR-17-0211 29180536 PMC6004321

[61] Majzner RG Ramakrishna S Yeom KW Patel S Chinnasamy H Schultz LM. GD2-CAR T cell therapy for H3K27M-mutated diffuse midline gliomas. Nature (2022) 603(7903):934–41. doi: 10.1038/s41586-022-04489-4 PMC8967714 35130560

[62] Levy PL Gros A. Fast track to personalized TCR T cell therapies. Cancer Cell (2022) 40(5):447–9. doi: 10.1016/j.ccell.2022.04.013 35537408

[63] Wilkie S van Schalkwyk MC Hobbs S Davies DM van der Stegen SJ Pereira AC. Dual targeting of ErbB2 and MUC1 in breast cancer using chimeric antigen receptors engineered to provide complementary signaling. J Clin Immunol (2012) 32(5):1059–70. doi: 10.1007/s10875-012-9689-9 22526592

[64] Kloss CC Condomines M Cartellieri M Bachmann M Sadelain M. Combinatorial antigen recognition with balanced signaling promotes selective tumor eradication by engineered T cells. Nat Biotechnol (2013) 31(1):71–5. doi: 10.1038/nbt.2459 PMC5505184 23242161

[65] Fedorov VD Themeli M Sadelain M. PD-1- and CTLA-4-based inhibitory chimeric antigen receptors (iCARs) divert off-target immunotherapy responses. Sci Transl Med (2013) 5(215):215ra172. doi: 10.1126/scitranslmed.3006597 PMC4238416 24337479

[66] Srivastava S Salter AI Liggitt D Yechan-Gunja S Sarvothama M Cooper K. Logic-gated ROR1 chimeric antigen receptor expression rescues T cell-mediated toxicity to normal tissues and enables selective tumor targeting. Cancer Cell (2019) 35(3):489–503.e8. doi: 10.1016/j.ccell.2019.02.003 30889382 PMC6450658

[67] Flugel CL Majzner RG Krenciute G Dotti G Riddell SR Wagner DL. Overcoming on-target, off-tumour toxicity of CAR T cell therapy for solid tumours. Nat Rev Clin Oncol (2023) 20(1):49–62. doi: 10.1038/s41571-022-00704-3 36418477 PMC10278599

[68] Tousley AM Rotiroti MC Labanieh L Rysavy LW Kim WJ Lareau C. Co-opting signalling molecules enables logic-gated control of CAR T cells. Nature (2023) 615(7952):507–16. doi: 10.1038/s41586-023-05778-2 PMC10564584 36890224

[69] Caruana I Weber G Ballard BC Wood MS Savoldo B Dotti G. K562-derived whole-cell vaccine enhances antitumor responses of CAR-redirected virus-specific cytotoxic T lymphocytes in vivo. Clin Cancer Res (2015) 21(13):2952–62. doi: 10.1158/1078-0432.CCR-14-2998 PMC4490027 25691731

[70] Tanaka M Tashiro H Omer B Lapteva N Ando J Ngo M. Vaccination targeting native receptors to enhance the function and proliferation of chimeric antigen receptor (CAR)-modified T cells. Clin Cancer Res (2017) 23(14):3499–509. doi: 10.1158/1078-0432.CCR-16-2138 PMC5511585 28183713

[71] Evgin L Kottke T Tonne J Thompson J Huff AL van Vloten J. Oncolytic virus-mediated expansion of dual-specific CAR T cells improves efficacy against solid tumors in mice. Sci Transl Med (2022) 14(640):eabn2231. doi: 10.1126/scitranslmed.abn2231 35417192 PMC9297825

[72] Liu E Marin D Banerjee P Macapinlac HA Thompson P Basar R. Use of CAR-transduced natural killer cells in CD19-positive lymphoid tumors. N Engl J Med (2020) 382(6):545–53. doi: 10.1056/NEJMoa1910607 PMC7101242 32023374

[73] Makkouk A Yang XC Barca T Lucas A Turkoz M Wong JTS. Off-the-shelf Vdelta1 gamma delta T cells engineered with glypican-3 (GPC-3)-specific chimeric antigen receptor (CAR) and soluble IL-15 display robust antitumor efficacy against hepatocellular carcinoma. J Immunother Cancer (2021) 9(12):e003441. doi: 10.1136/jitc-2021-003441 34916256 PMC8679077

[74] Simonetta F Lohmeyer JK Hirai T Maas-Bauer K Alvarez M Wenokur AS. Allogeneic CAR invariant natural killer T cells exert potent antitumor effects through host CD8 T-cell cross-priming. Clin Cancer Res (2021) 27(21):6054–64. doi: 10.1158/1078-0432.CCR-21-1329 PMC8563377 34376537

[75] Crowther MD Dolton G Legut M Caillaud ME Lloyd A Attaf M. Genome-wide CRISPR-Cas9 screening reveals ubiquitous T cell cancer targeting *via* the monomorphic MHC class I-related protein MR1. Nat Immunol (2020) 21(2):178–85. doi: 10.1038/s41590-019-0578-8 PMC6983325 31959982

[76] Reinhard K Rengstl B Oehm P Michel K Billmeier A Hayduk N. An RNA vaccine drives expansion and efficacy of claudin-CAR-T cells against solid tumors. Science (2020) 367(6476):446–53. doi: 10.1126/science.aay5967 31896660

[77] Ma L Dichwalkar T Chang JYH Cossette B Garafola D Zhang AQ. Enhanced CAR-T cell activity against solid tumors by vaccine boosting through the chimeric receptor. Science (2019) 365(6449):162–8. doi: 10.1126/science.aav8692 PMC6800571 31296767

[78] Ma L Hostetler A Morgan DM Maiorino L Sulkaj I Whittaker CA. Vaccine-boosted CAR T crosstalk with host immunity to reject tumors with antigen heterogeneity. Cell (2023). doi: 10.1136/jitc-2023-SITC2023.0286 PMC10372881 37413990

[79] Vaday GG Franitza S Schor H Hecht I Brill A Cahalon L. Combinatorial signals by inflammatory cytokines and chemokines mediate leukocyte interactions with extracellular matrix. J Leukoc Biol (2001) 69(6):885–92. doi: 10.1189/jlb.69.6.885 11404372

[80] Ward SG Westwick J. Chemokines: understanding their role in T-lymphocyte biology. Biochem J (1998) 333(Pt 3):457–70. doi: 10.1042/bj3330457 PMC1219606 9677302

[81] Adachi K Kano Y Nagai T Okuyama N Sakoda Y Tamada K. IL-7 and CCL19 expression in CAR-T cells improves immune cell infiltration and CAR-T cell survival in the tumor. Nat Biotechnol (2018) 36(4):346–51. doi: 10.1038/nbt.4086 29505028

[82] Rapp M Grassmann S Chaloupka M Layritz P Kruger S Ormanns S. C-C chemokine receptor type-4 transduction of T cells enhances interaction with dendritic cells, tumor infiltration and therapeutic efficacy of adoptive T cell transfer. Oncoimmunology (2016) 5(3):e1105428. doi: 10.1080/2162402X.2015.1105428 27195186 PMC4859768

[83] Garetto S Sardi C Martini E Roselli G Morone D Angioni R. Tailored chemokine receptor modification improves homing of adoptive therapy T cells in a spontaneous tumor model. Oncotarget (2016) 7(28):43010–26. doi: 10.18632/oncotarget.9280 PMC5190004 27177227

[84] Siddiqui I Erreni M van Brakel M Debets R Allavena P. Enhanced recruitment of genetically modified CX3CR1-positive human T cells into Fractalkine/CX3CL1 expressing tumors: importance of the chemokine gradient. J Immunother Cancer (2016) 4:21. doi: 10.1186/s40425-016-0125-1 27096098 PMC4836203

[85] Muller N Michen S Tietze S Topfer K Schulte A Lamszus K. Engineering NK cells modified with an EGFRvIII-specific chimeric antigen receptor to overexpress CXCR4 improves immunotherapy of CXCL12/SDF-1alpha-secreting glioblastoma. J Immunother (2015) 38(5):197–210. doi: 10.1097/CJI.0000000000000082 25962108 PMC4428685

[86] Moon EK Carpenito C Sun J Wang LC Kapoor V Predina J. Expression of a functional CCR2 receptor enhances tumor localization and tumor eradication by retargeted human T cells expressing a mesothelin-specific chimeric antibody receptor. Clin Cancer Res (2011) 17(14):4719–30. doi: 10.1158/1078-0432.CCR-11-0351 PMC3612507 21610146

[87] Peng W Ye Y Rabinovich BA Liu C Lou Y Zhang M. Transduction of tumor-specific T cells with CXCR2 chemokine receptor improves migration to tumor and antitumor immune responses. Clin Cancer Res (2010) 16(22):5458–68. doi: 10.1158/1078-0432.CCR-10-0712 PMC3476703 20889916

[88] Li K Zhu Z Luo J Fang J Zhou H Hu M. Impact of chemokine receptor CXCR3 on tumor-infiltrating lymphocyte recruitment associated with favorable prognosis in advanced gastric cancer. Int J Clin Exp Pathol (2015) 8(11):14725–32. PMC4713583 26823797

[89] Liu G Rui W Zheng H Huang D Yu F Zhang Y. CXCR2-modified CAR-T cells have enhanced trafficking ability that improves treatment of hepatocellular carcinoma. Eur J Immunol (2020) 50(5):712–24. doi: 10.1002/eji.201948457 31981231

[90] Jin L Tao H Karachi A Long Y Hou AY Na M. CXCR1- or CXCR2-modified CAR T cells co-opt IL-8 for maximal antitumor efficacy in solid tumors. Nat Commun (2019) 10(1):4016. doi: 10.1038/s41467-019-11869-4 31488817 PMC6728370

[91] Ng YY Tay JCK Wang S. CXCR1 expression to improve anti-cancer efficacy of intravenously injected CAR-NK cells in mice with peritoneal xenografts. Mol Ther Oncolytics (2020) 16:75–85. doi: 10.1016/j.omto.2019.12.006 31970285 PMC6965500

[92] Wang Y Wang J Yang X Yang J Lu P Zhao L. Chemokine receptor CCR2b enhanced anti-tumor function of chimeric antigen receptor T cells targeting mesothelin in a non-small-cell lung carcinoma model. Front Immunol (2021) 12:628906. doi: 10.3389/fimmu.2021.628906 33777013 PMC7992009

[93] Lesch S Blumenberg V Stoiber S Gottschlich A Ogonek J Cadilha BL. T cells armed with C-X-C chemokine receptor type 6 enhance adoptive cell therapy for pancreatic tumours. Nat BioMed Eng (2021) 5(11):1246–60. doi: 10.1038/s41551-021-00737-6 PMC7611996 34083764

[94] Watson HA Durairaj RRP Ohme J Alatsatianos M Almutairi H Mohammed RN. L-selectin enhanced T cells improve the efficacy of cancer immunotherapy. Front Immunol (2019) 10:1321. doi: 10.3389/fimmu.2019.01321 31249570 PMC6582763

[95] Jung IY Noguera-Ortega E Bartoszek R Collins SM Williams E Davis M. Tissue-resident memory CAR T cells with stem-like characteristics display enhanced efficacy against solid and liquid tumors. Cell Rep Med (2023) 4(6):101053. doi: 10.1016/j.xcrm.2023.101053 37224816 PMC10313923

[96] Milner JJ Toma C Yu B Zhang K Omilusik K Phan AT. Runx3 programs CD8(+) T cell residency in non-lymphoid tissues and tumours. Nature (2017) 552(7684):253–7. doi: 10.1038/nature24993 PMC5747964 29211713

[97] Leidner R Sanjuan Silva N Huang H Sprott D Zheng C Shih YP. Neoantigen T-cell receptor gene therapy in pancreatic cancer. N Engl J Med (2022) 386(22):2112–9. doi: 10.1056/NEJMoa2119662 PMC9531755 35648703

[98] Nishio N Diaconu I Liu H Cerullo V Caruana I Hoyos V. Armed oncolytic virus enhances immune functions of chimeric antigen receptor-modified T cells in solid tumors. Cancer Res (2014) 74(18):5195–205. doi: 10.1158/0008-5472.CAN-14-0697 PMC4167556 25060519

[99] Moon EK Wang LS Bekdache K Lynn RC Lo A Thorne SH. Intra-tumoral delivery of CXCL11 *via* a vaccinia virus, but not by modified T cells, enhances the efficacy of adoptive T cell therapy and vaccines. Oncoimmunology (2018) 7(3):e1395997. doi: 10.1080/2162402X.2017.1395997 29399394 PMC5790399

[100] Rosewell Shaw A Porter CE Watanabe N Tanoue K Sikora A Gottschalk S. Adenovirotherapy delivering cytokine and checkpoint inhibitor augments CAR T cells against metastatic head and neck cancer. Mol Ther (2017) 25(11):2440–51. doi: 10.1016/j.ymthe.2017.09.010 PMC5675597 28974431

[101] Tanoue K Rosewell Shaw A Watanabe N Porter C Rana B Gottschalk S. Armed oncolytic adenovirus-expressing PD-L1 mini-body enhances antitumor effects of chimeric antigen receptor T cells in solid tumors. Cancer Res (2017) 77(8):2040–51. doi: 10.1158/0008-5472.CAN-16-1577 PMC5392365 28235763

[102] Watanabe K Luo Y Da T Guedan S Ruella M Scholler J. Pancreatic cancer therapy with combined mesothelin-redirected chimeric antigen receptor T cells and cytokine-armed oncolytic adenoviruses. JCI Insight (2018) 3(7):e99573. doi: 10.1172/jci.insight.99573 29618658 PMC5928866

[103] Evgin L Huff AL Wongthida P Thompson J Kottke T Tonne J. Oncolytic virus-derived type I interferon restricts CAR T cell therapy. Nat Commun (2020) 11(1):3187. doi: 10.1038/s41467-020-17011-z 32581235 PMC7314766

[104] Evgin L Vile RG. Parking CAR T cells in tumours: oncolytic viruses as valets or vandals? Cancers (Basel) (2021) 13(5):1106. doi: 10.3390/cancers13051106 33807553 PMC7961585

[105] Ajina A Maher J. Synergistic combination of oncolytic virotherapy with CAR T-cell therapy. Prog Mol Biol Transl Sci (2019) 164:217–92. doi: 10.1016/bs.pmbts.2019.06.015 31383406

[106] Provenzano PP Cuevas C Chang AE Goel VK Von Hoff DD Hingorani SR. Enzymatic targeting of the stroma ablates physical barriers to treatment of pancreatic ductal adenocarcinoma. Cancer Cell (2012) 21(3):418–29. doi: 10.1016/j.ccr.2012.01.007 PMC3371414 22439937

[107] Kakarla S Chow KK Mata M Shaffer DR Song XT Wu MF. Antitumor effects of chimeric receptor engineered human T cells directed to tumor stroma. Mol Ther (2013) 21(8):1611–20. doi: 10.1038/mt.2013.110 PMC3734659 23732988

[108] Schuberth PC Hagedorn C Jensen SM Gulati P van den Broek M Mischo A. Treatment of Malignant pleural mesothelioma by fibroblast activation protein-specific re-directed T cells. J Transl Med (2013) 11:187. doi: 10.1186/1479-5876-11-187 23937772 PMC3751305

[109] Lo A Wang LS Scholler J Monslow J Avery D Newick K. Tumor-promoting desmoplasia is disrupted by depleting FAP-expressing stromal cells. Cancer Res (2015) 75(14):2800–10. doi: 10.1158/0008-5472.CAN-14-3041 PMC4506263 25979873

[110] Roberts EW Deonarine A Jones JO Denton AE Feig C Lyons SK. Depletion of stromal cells expressing fibroblast activation protein-alpha from skeletal muscle and bone marrow results in cachexia and anemia. J Exp Med (2013) 210(6):1137–51. doi: 10.1084/jem.20122344 PMC3674708 23712428

[111] Tran E Chinnasamy D Yu Z Morgan RA Lee CC Restifo NP. Immune targeting of fibroblast activation protein triggers recognition of multipotent bone marrow stromal cells and cachexia. J Exp Med (2013) 210(6):1125–35. doi: 10.1084/jem.20130110 PMC3674706 23712432

[112] Zhao R Cui Y Zheng Y Li S Lv J Wu Q. Human hyaluronidase PH20 potentiates the antitumor activities of mesothelin-specific CAR-T cells against gastric cancer. Front Immunol (2021) 12:660488. doi: 10.3389/fimmu.2021.660488 34326835 PMC8313856

[113] Caruana I Savoldo B Hoyos V Weber G Liu H Kim ES. Heparanase promotes tumor infiltration and antitumor activity of CAR-redirected T lymphocytes. Nat Med (2015) 21(5):524–9. doi: 10.1038/nm.3833 PMC4425589 25849134

[114] Rodriguez-Garcia A Lynn RC Poussin M Eiva MA Shaw LC O'Connor RS. CAR-T cell-mediated depletion of immunosuppressive tumor-associated macrophages promotes endogenous antitumor immunity and augments adoptive immunotherapy. Nat Commun (2021) 12(1):877. doi: 10.1038/s41467-021-20893-2 33563975 PMC7873057

[115] Nalawade SA Shafer P Bajgain P McKenna MK Ali A Kelly L. Selectively targeting myeloid-derived suppressor cells through TRAIL receptor 2 to enhance the efficacy of CAR T cell therapy for treatment of breast cancer. J Immunother Cancer (2021) 9(11):e003237. doi: 10.1136/jitc-2021-003237 34815355 PMC8611441

[116] Condamine T Kumar V Ramachandran IR Youn JI Celis E Finnberg N. ER stress regulates myeloid-derived suppressor cell fate through TRAIL-R-mediated apoptosis. J Clin Invest (2014) 124(6):2626–39. doi: 10.1172/JCI74056 PMC4038578 24789911

[117] Dominguez GA Condamine T Mony S Hashimoto A Wang F Liu Q. Selective targeting of myeloid-derived suppressor cells in cancer patients using DS-8273a, an agonistic TRAIL-R2 antibody. Clin Cancer Res (2017) 23(12):2942–50. doi: 10.1158/1078-0432.CCR-16-1784 PMC5468499 27965309

[118] Chandler C Liu T Buckanovich R Coffman LG. The double edge sword of fibrosis in cancer. Transl Res (2019) 209:55–67. doi: 10.1016/j.trsl.2019.02.006 30871956 PMC6545239

[119] Levental KR Yu H Kass L Lakins JN Egeblad M Erler JT. Matrix crosslinking forces tumor progression by enhancing integrin signaling. Cell (2009) 139(5):891–906. doi: 10.1016/j.cell.2009.10.027 19931152 PMC2788004

[120] Piersma B Bank RA. Collagen cross-linking mediated by lysyl hydroxylase 2: an enzymatic battlefield to combat fibrosis. Essays Biochem (2019) 63(3):377–87. doi: 10.1042/EBC20180051 31324706

[121] Hosein AN Brekken RA Maitra A. Pancreatic cancer stroma: an update on therapeutic targeting strategies. Nat Rev Gastroenterol Hepatol (2020) 17(8):487–505. doi: 10.1038/s41575-020-0300-1 32393771 PMC8284850

[122] Sun X Wu B Chiang HC Deng H Zhang X Xiong W. Tumour DDR1 promotes collagen fibre alignment to instigate immune exclusion. Nature (2021) 599(7886):673–8. doi: 10.1038/s41586-021-04057-2 PMC8839149 34732895

[123] Murad JP Tilakawardane D Park AK Lopez LS Young CA Gibson J. Pre-conditioning modifies the TME to enhance solid tumor CAR T cell efficacy and endogenous protective immunity. Mol Ther (2021) 29(7):2335–49. doi: 10.1016/j.ymthe.2021.02.024 PMC8261088 33647456

[124] Kolb M Kirschner J Riedel W Wirtz H Schmidt M. Cyclophosphamide pulse therapy in idiopathic pulmonary fibrosis. Eur Respir J (1998) 12(6):1409–14. doi: 10.1183/09031936.98.12061409 9877500

[125] Qi C Gong J Li J Liu D Qin Y Ge S. Claudin18.2-specific CAR T cells in gastrointestinal cancers: phase 1 trial interim results. Nat Med (2022) 28(6):1189–98. doi: 10.1038/s41591-022-01800-8 PMC9205778 35534566

[126] Uslu U Da T Assenmacher CA Scholler J Young RM Tchou J. Chimeric antigen receptor T cells as adjuvant therapy for unresectable adenocarcinoma. Sci Adv (2023) 9(2):eade2526. doi: 10.1126/sciadv.ade2526 36630514 PMC9833675

[127] Lanitis E Dangaj D Irving M Coukos G. Mechanisms regulating T-cell infiltration and activity in solid tumors. Ann Oncol (2017) 28(suppl_12):xii18–32. doi: 10.1093/annonc/mdx238 29045511

[128] Hawkins ER D'Souza RR Klampatsa A. Armored CAR T-cells: the next chapter in T-cell cancer immunotherapy. Biologics (2021) 15:95–105. doi: 10.2147/BTT.S291768 33883875 PMC8053711

[129] Chang CH Qiu J O'Sullivan D Buck MD Noguchi T Curtis JD. Metabolic competition in the tumor microenvironment is a driver of cancer progression. Cell (2015) 162(6):1229–41. doi: 10.1016/j.cell.2015.08.016 PMC4864363 26321679

[130] Peng M Yin N Chhangawala S Xu K Leslie CS Li MO. Aerobic glycolysis promotes T helper 1 cell differentiation through an epigenetic mechanism. Science (2016) 354(6311):481–4. doi: 10.1126/science.aaf6284 PMC5539971 27708054

[131] Hermans D Gautam S Garcia-Canaveras JC Gromer D Mitra S Spolski R. Lactate dehydrogenase inhibition synergizes with IL-21 to promote CD8(+) T cell stemness and antitumor immunity. Proc Natl Acad Sci U S A (2020) 117(11):6047–55. doi: 10.1073/pnas.1920413117 PMC7084161 32123114

[132] Papandreou I Cairns RA Fontana L Lim AL Denko NC. HIF-1 mediates adaptation to hypoxia by actively downregulating mitochondrial oxygen consumption. Cell Metab (2006) 3(3):187–97. doi: 10.1016/j.cmet.2006.01.012 16517406

[133] Seagroves TN Ryan HE Lu H Wouters BG Knapp M Thibault P. Transcription factor HIF-1 is a necessary mediator of the pasteur effect in mammalian cells. Mol Cell Biol (2001) 21(10):3436–44. doi: 10.1128/MCB.21.10.3436-3444.2001 PMC100265 11313469

[134] Palazon A Tyrakis PA Macias D Velica P Rundqvist H Fitzpatrick S. An HIF-1alpha/VEGF-A axis in cytotoxic T cells regulates tumor progression. Cancer Cell (2017) 32(5):669–83.e5. doi: 10.1016/j.ccell.2017.10.003 29136509 PMC5691891

[135] Geiger R Rieckmann JC Wolf T Basso C Feng Y Fuhrer T. L-arginine modulates T cell metabolism and enhances survival and anti-tumor activity. Cell (2016) 167(3):829–42.e13. doi: 10.1016/j.cell.2016.09.031 27745970 PMC5075284

[136] Cimen Bozkus C Elzey BD Crist SA Ellies LG Ratliff TL. Expression of cationic amino acid transporter 2 is required for myeloid-derived suppressor cell-mediated control of T cell immunity. J Immunol (2015) 195(11):5237–50. doi: 10.4049/jimmunol.1500959 PMC4655170 26491198

[137] Halaby MJ McGaha TL. Amino acid transport and metabolism in myeloid function. Front Immunol (2021) 12:695238. doi: 10.3389/fimmu.2021.695238 34456909 PMC8397459

[138] Zimmermann H. Extracellular metabolism of ATP and other nucleotides. Naunyn Schmiedebergs Arch Pharmacol (2000) 362(4-5):299–309. doi: 10.1007/s002100000309 11111825

[139] Vigano S Alatzoglou D Irving M Menetrier-Caux C Caux C Romero P. Targeting adenosine in cancer immunotherapy to enhance T-cell function. Front Immunol (2019) 10:925. doi: 10.3389/fimmu.2019.00925 31244820 PMC6562565

[140] Beavis PA Henderson MA Giuffrida L Mills JK Sek K Cross RS. Targeting the adenosine 2A receptor enhances chimeric antigen receptor T cell efficacy. J Clin Invest (2017) 127(3):929–41. doi: 10.1172/JCI89455 PMC5330718 28165340

[141] Giuffrida L Sek K Henderson MA Lai J Chen AXY Meyran D. CRISPR/Cas9 mediated deletion of the adenosine A2A receptor enhances CAR T cell efficacy. Nat Commun (2021) 12(1):3236. doi: 10.1038/s41467-021-23331-5 34050151 PMC8163771

[142] Seifert M Benmebarek M-R Briukhovetska D Märkl F Dörr J Cadilha BL. Impact of the selective A2AR and A2BR dual antagonist AB928/etrumadenant on CAR T cell function. Br J Cancer (2022) 127(12):2175–85. doi: 10.1038/s41416-022-02013-z PMC9726885 36266575

[143] Deaglio S Dwyer KM Gao W Friedman D Usheva A Erat A. Adenosine generation catalyzed by CD39 and CD73 expressed on regulatory T cells mediates immune suppression. J Exp Med (2007) 204(6):1257–65. doi: 10.1084/jem.20062512 PMC2118603 17502665

[144] Ohta A Kini R Ohta A Subramanian M Madasu M Sitkovsky M. The development and immunosuppressive functions of CD4(+) CD25(+) FoxP3(+) regulatory T cells are under influence of the adenosine-A2A adenosine receptor pathway. Front Immunol (2012) 3:190. doi: 10.3389/fimmu.2012.00190 22783261 PMC3389649

[145] Schneider E Winzer R Rissiek A Ricklefs I Meyer-Schwesinger C Ricklefs FL. CD73-mediated adenosine production by CD8 T cell-derived extracellular vesicles constitutes an intrinsic mechanism of immune suppression. Nat Commun (2021) 12(1):5911. doi: 10.1038/s41467-021-26134-w 34625545 PMC8501027

[146] Rissiek A Baumann I Cuapio A Mautner A Kolster M Arck PC. The expression of CD39 on regulatory T cells is genetically driven and further upregulated at sites of inflammation. J Autoimmun (2015) 58:12–20. doi: 10.1016/j.jaut.2014.12.007 25640206

[147] Mallikarjuna P Zhou Y Landstrom M. The synergistic cooperation between TGF-beta and hypoxia in cancer and fibrosis. Biomolecules (2022) 12(5):635. doi: 10.3390/biom12050635 35625561 PMC9138354

[148] Alishah K Birtel M Masoumi E Jafarzadeh L Mirzaee HR Hadjati J. CRISPR/Cas9-mediated TGFbetaRII disruption enhances anti-tumor efficacy of human chimeric antigen receptor T cells in vitro. J Transl Med (2021) 19(1):482. doi: 10.1186/s12967-021-03146-0 34838059 PMC8627098

[149] Bollard CM Tripic T Cruz CR Dotti G Gottschalk S Torrano V. Tumor-specific T-cells engineered to overcome tumor immune evasion induce clinical responses in patients with relapsed hodgkin lymphoma. J Clin Oncol (2018) 36(11):1128–39. doi: 10.1200/JCO.2017.74.3179 PMC5891126 29315015

[150] Chu NJ Overstreet MG Gilbreth R Clarke L Gesse C Tu E. Abstract 2837: Synthetic TGFb blockade preserves effector function and maintains stemness of GPC3 CAR-T against hepatocellular carcinoma. Cancer Res (2022) 82(12_Supplement):2837. doi: 10.1158/1538-7445.AM2022-2837

[151] Kloss CC Lee J Zhang A Chen F Melenhorst JJ Lacey SF. Dominant-negative TGF-beta receptor enhances PSMA-targeted human CAR T cell proliferation and augments prostate cancer eradication. Mol Ther (2018) 26(7):1855–66. doi: 10.1016/j.ymthe.2018.05.003 PMC6037129 29807781

[152] Li K Xu J Wang J Lu C Dai Y Dai Q. Dominant-negative transforming growth factor-beta receptor-armoured mesothelin-targeted chimeric antigen receptor T cells slow tumour growth in a mouse model of ovarian cancer. Cancer Immunol Immunother (2023) 72(4):917–28. doi: 10.1007/s00262-022-03290-6 PMC10025183 36166071

[153] Narayan V Barber-Rotenberg JS Jung IY Lacey SF Rech AJ Davis MM. PSMA-targeting TGFbeta-insensitive armored CAR T cells in metastatic castration-resistant prostate cancer: a phase 1 trial. Nat Med (2022) 28(4):724–34. doi: 10.1038/s41591-022-01726-1 PMC10308799 35314843

[154] Tang N Cheng C Zhang X Qiao M Li N Mu W. TGF-beta inhibition *via* CRISPR promotes the long-term efficacy of CAR T cells against solid tumors. JCI Insight (2020) 5(4):e133977. doi: 10.1172/jci.insight.133977 31999649 PMC7101140

[155] van Dyk D Zanvit P Fazenbaker C McGlinchey K Luo W Pezold J. Abstract LB085: Antitumor activity of AZD0754, a dnTGFbRII armored STEAP2 targeted CAR-T therapy, in preclinical models of prostate cancer. Cancer Res (2023) 83(8_Supplement):LB085–LB. doi: 10.1158/1538-7445.AM2023-LB085 PMC10645390 37966111

[156] Zhang N Bevan MJ. TGF-beta signaling to T cells inhibits autoimmunity during lymphopenia-driven proliferation. Nat Immunol (2012) 13(7):667–73. doi: 10.1038/ni.2319 PMC3380154 22634866

[157] Frangogiannis N. Transforming growth factor-beta in tissue fibrosis. J Exp Med (2020) 217(3):e20190103. doi: 10.1084/jem.20190103 32997468 PMC7062524

[158] Cherkassky L Morello A Villena-Vargas J Feng Y Dimitrov DS Jones DR. Human CAR T cells with cell-intrinsic PD-1 checkpoint blockade resist tumor-mediated inhibition. J Clin Invest (2016) 126(8):3130–44. doi: 10.1172/JCI83092 PMC4966328 27454297

[159] Liu X Ranganathan R Jiang S Fang C Sun J Kim S. A chimeric switch-receptor targeting PD1 augments the efficacy of second-generation CAR T cells in advanced solid tumors. Cancer Res (2016) 76(6):1578–90. doi: 10.1158/0008-5472.CAN-15-2524 PMC4800826 26979791

[160] Prosser ME Brown CE Shami AF Forman SJ Jensen MC. Tumor PD-L1 co-stimulates primary human CD8(+) cytotoxic T cells modified to express a PD1:CD28 chimeric receptor. Mol Immunol (2012) 51(3-4):263–72. doi: 10.1016/j.molimm.2012.03.023 22503210

[161] Yamamoto TN Lee PH Vodnala SK Gurusamy D Kishton RJ Yu Z. T cells genetically engineered to overcome death signaling enhance adoptive cancer immunotherapy. J Clin Invest (2019) 129(4):1551–65. doi: 10.1172/JCI121491 PMC6436880 30694219

[162] Zhang Y Zhang X Cheng C Mu W Liu X Li N. CRISPR-Cas9 mediated LAG-3 disruption in CAR-T cells. Front Med (2017) 11(4):554–62. doi: 10.1007/s11684-017-0543-6 28625015

[163] Rowshanravan B Halliday N Sansom DM. CTLA-4: a moving target in immunotherapy. Blood (2018) 131(1):58–67. doi: 10.1182/blood-2017-06-741033 29118008 PMC6317697

[164] Liu Y Di S Shi B Zhang H Wang Y Wu X. Armored inducible expression of IL-12 enhances antitumor activity of glypican-3-targeted chimeric antigen receptor-engineered T cells in hepatocellular carcinoma. J Immunol (2019) 203(1):198–207. doi: 10.4049/jimmunol.1800033 31142602

[165] Yeku OO Purdon TJ Koneru M Spriggs D Brentjens RJ. Armored CAR T cells enhance antitumor efficacy and overcome the tumor microenvironment. Sci Rep (2017) 7(1):10541. doi: 10.1038/s41598-017-10940-8 28874817 PMC5585170

[166] Zhang L Davies JS Serna C Yu Z Restifo NP Rosenberg SA. Enhanced efficacy and limited systemic cytokine exposure with membrane-anchored interleukin-12 T-cell therapy in murine tumor models. J Immunother Cancer (2020) 8(1):e000210. doi: 10.1136/jitc-2019-000210 31959727 PMC7057422

[167] Rafiq S Hackett CS Brentjens RJ. Engineering strategies to overcome the current roadblocks in CAR T cell therapy. Nat Rev Clin Oncol (2020) 17(3):147–67. doi: 10.1038/s41571-019-0297-y PMC7223338 31848460

[168] Hu B Ren J Luo Y Keith B Young RM Scholler J. Augmentation of antitumor immunity by human and mouse CAR T cells secreting IL-18. Cell Rep (2017) 20(13):3025–33. doi: 10.1016/j.celrep.2017.09.002 PMC6002762 28954221

[169] Avanzi MP G van Leeuwen D Li X Cheung K Park H Purdon TJ. IL-18 secreting CAR T cells enhance cell persistence, induce prolonged B cell aplasia and eradicate CD19+ Tumor cells without need for prior conditioning. Blood (2016) 128(22):816–. doi: 10.1182/blood.V128.22.816.816

[170] Pegram HJ Lee JC Hayman EG Imperato GH Tedder TF Sadelain M. Tumor-targeted T cells modified to secrete IL-12 eradicate systemic tumors without need for prior conditioning. Blood (2012) 119(18):4133–41. doi: 10.1182/blood-2011-12-400044 PMC3359735 22354001

[171] Zhou LJ Smith HM Waldschmidt TJ Schwarting R Daley J Tedder TF. Tissue-specific expression of the human CD19 gene in transgenic mice inhibits antigen-independent B-lymphocyte development. Mol Cell Biol (1994) 14(6):3884–94. doi: 10.1128/MCB.14.6.3884 PMC358755 7515149

[172] Svoboda J Landsburg DL Chong EA Barta SK Nasta SD Ruella M. Fourth generation hucart19-il18 produces durable responses in lymphoma patients previously relapsed/refractory to anti-cd19 car T-cell therapy. Hematological Oncol (2023) 41(S2):35–7. doi: 10.1002/hon.3163_6

[173] Chen Y Sun C Landoni E Metelitsa L Dotti G Savoldo B. Eradication of neuroblastoma by T cells redirected with an optimized GD2-specific chimeric antigen receptor and interleukin-15. Clin Cancer Res (2019) 25(9):2915–24. doi: 10.1158/1078-0432.CCR-18-1811 30617136

[174] Hoyos V Savoldo B Quintarelli C Mahendravada A Zhang M Vera J. Engineering CD19-specific T lymphocytes with interleukin-15 and a suicide gene to enhance their anti-lymphoma/leukemia effects and safety. Leukemia (2010) 24(6):1160–70. doi: 10.1038/leu.2010.75 PMC2888148 20428207

[175] Krenciute G Prinzing BL Yi Z Wu MF Liu H Dotti G. Transgenic expression of IL15 improves antiglioma activity of IL13Ralpha2-CAR T cells but results in antigen loss variants. Cancer Immunol Res (2017) 5(7):571–81. doi: 10.1158/2326-6066.CIR-16-0376 PMC5746871 28550091

[176] Fehniger TA Suzuki K Ponnappan A VanDeusen JB Cooper MA Florea SM. Fatal leukemia in interleukin 15 transgenic mice follows early expansions in natural killer and memory phenotype CD8+ T cells. J Exp Med (2001) 193(2):219–31. doi: 10.1084/jem.193.2.219 PMC2193336 11208862

[177] Mantovani A Allavena P Marchesi F Garlanda C. Macrophages as tools and targets in cancer therapy. Nat Rev Drug Discovery (2022) 21(11):799–820. doi: 10.1038/s41573-022-00520-5 35974096 PMC9380983

[178] Ruella M Klichinsky M Kenderian SS Shestova O Ziober A Kraft DO. Overcoming the immunosuppressive tumor microenvironment of hodgkin lymphoma using chimeric antigen receptor T cells. Cancer Discovery (2017) 7(10):1154–67. doi: 10.1158/2159-8290.CD-16-0850 PMC5628114 28576927

[179] Dehbashi M Hojati Z Motovali-Bashi M Ganjalikhany MR Cho WC Shimosaka A. A novel CAR expressing NK cell targeting CD25 with the prospect of overcoming immune escape mechanism in cancers. Front Oncol (2021) 11:649710. doi: 10.3389/fonc.2021.649710 34055618 PMC8160382

[180] Onda M Kobayashi K Pastan I. Depletion of regulatory T cells in tumors with an anti-CD25 immunotoxin induces CD8 T cell-mediated systemic antitumor immunity. Proc Natl Acad Sci U S A (2019) 116(10):4575–82. doi: 10.1073/pnas.1820388116 PMC6410866 30760587

[181] Melenhorst JJ Chen GM Wang M Porter DL Chen C Collins MA. Decade-long leukaemia remissions with persistence of CD4(+) CAR T cells. Nature (2022) 602(7897):503–9. doi: 10.1038/s41586-021-04390-6 PMC9166916 35110735

[182] Soerens AG Kunzli M Quarnstrom CF Scott MC Swanson L Locquiao JJ. Functional T cells are capable of supernumerary cell division and longevity. Nature (2023) 614(7949):762–6. doi: 10.1038/s41586-022-05626-9 PMC11617068 36653453

[183] Yaguchi T Kobayashi A Inozume T Morii K Nagumo H Nishio H. Human PBMC-transferred murine MHC class I/II-deficient NOG mice enable long-term evaluation of human immune responses. Cell Mol Immunol (2018) 15(11):953–62. doi: 10.1038/cmi.2017.106 PMC6207709 29151581

[184] Brehm MA Kenney LL Wiles MV Low BE Tisch RM Burzenski L. Lack of acute xenogeneic graft- versus-host disease, but retention of T-cell function following engraftment of human peripheral blood mononuclear cells in NSG mice deficient in MHC class I and II expression. FASEB J (2019) 33(3):3137–51. doi: 10.1096/fj.201800636R PMC6404556 30383447

[185] Petersdorf EW. Role of major histocompatibility complex variation in graft-versus-host disease after hematopoietic cell transplantation. F1000Res (2017) 6:617. doi: 10.12688/f1000research.10990.1 28529723 PMC5419254

[186] Kato D Yaguchi T Iwata T Katoh Y Morii K Tsubota K. GPC1 specific CAR-T cells eradicate established solid tumor without adverse effects and synergize with anti-PD-1 Ab. Elife (2020) 9:e49392. doi: 10.7554/eLife.49392 32228854 PMC7108862

[187] Kawalekar OU OC RS Fraietta JA Guo L McGettigan SE Posey AD Jr.. Distinct signaling of coreceptors regulates specific metabolism pathways and impacts memory development in CAR T cells. Immunity (2016) 44(3):712. doi: 10.1016/j.immuni.2016.01.021 28843072

[188] Brentjens RJ Davila ML Riviere I Park J Wang X Cowell LG. CD19-targeted T cells rapidly induce molecular remissions in adults with chemotherapy-refractory acute lymphoblastic leukemia. Sci Transl Med (2013) 5(177):177ra38. doi: 10.1126/scitranslmed.3005930 PMC3742551 23515080

[189] Lee DW Kochenderfer JN Stetler-Stevenson M Cui YK Delbrook C Feldman SA. T cells expressing CD19 chimeric antigen receptors for acute lymphoblastic leukaemia in children and young adults: a phase 1 dose-escalation trial. Lancet (2015) 385(9967):517–28. doi: 10.1016/S0140-6736(14)61403-3 PMC7065359 25319501

[190] Porter DL Hwang WT Frey NV Lacey SF Shaw PA Loren AW. Chimeric antigen receptor T cells persist and induce sustained remissions in relapsed refractory chronic lymphocytic leukemia. Sci Transl Med (2015) 7(303):303ra139. doi: 10.1126/scitranslmed.aac5415 PMC5909068 26333935

[191] Pearce EL Walsh MC Cejas PJ Harms GM Shen H Wang LS. Enhancing CD8 T-cell memory by modulating fatty acid metabolism. Nature (2009) 460(7251):103–7. doi: 10.1038/nature08097 PMC2803086 19494812

[192] Sadelain M Brentjens R Riviere I. The basic principles of chimeric antigen receptor design. Cancer Discovery (2013) 3(4):388–98. doi: 10.1158/2159-8290.CD-12-0548 PMC3667586 23550147

[193] Dotti G Gottschalk S Savoldo B Brenner MK. Design and development of therapies using chimeric antigen receptor-expressing T cells. Immunol Rev (2014) 257(1):107–26. doi: 10.1111/imr.12131 PMC3874724 24329793

[194] Tammana S Huang X Wong M Milone MC Ma L Levine BL. 4-1BB and CD28 signaling plays a synergistic role in redirecting umbilical cord blood T cells against B-cell Malignancies. Hum Gene Ther (2010) 21(1):75–86. doi: 10.1089/hum.2009.122 19719389 PMC2861957

[195] Guedan S Posey AD Jr. Shaw C Wing A Da T Patel PR. Enhancing CAR T cell persistence through ICOS and 4-1BB costimulation. JCI Insight (2018) 3(1):e96976. doi: 10.1172/jci.insight.96976 29321369 PMC5821198

[196] Enblad G Karlsson H Gammelgard G Wenthe J Lovgren T Amini RM. A phase I/IIa trial using CD19-targeted third-generation CAR T cells for lymphoma and leukemia. Clin Cancer Res (2018) 24(24):6185–94. doi: 10.1158/1078-0432.CCR-18-0426 30097433

[197] Zhao X Yang J Zhang X Lu XA Xiong M Zhang J. Efficacy and safety of CD28- or 4-1BB-based CD19 CAR-T cells in B cell acute lymphoblastic leukemia. Mol Ther Oncolytics (2020) 18:272–81. doi: 10.1016/j.omto.2020.06.016 PMC7378699 32728615

[198] Rosenberg SA. IL-2: the first effective immunotherapy for human cancer. J Immunol (2014) 192(12):5451–8. doi: 10.4049/jimmunol.1490019 PMC6293462 24907378

[199] Aspuria PJ Vivona S Bauer M Semana M Ratti N McCauley S. An orthogonal IL-2 and IL-2Rbeta system drives persistence and activation of CAR T cells and clearance of bulky lymphoma. Sci Transl Med (2021) 13(625):eabg7565. doi: 10.1126/scitranslmed.abg7565 34936383

[200] Zhang Q Hresko ME Picton LK Su L Hollander MJ Nunez-Cruz S. A human orthogonal IL-2 and IL-2Rbeta system enhances CAR T cell expansion and antitumor activity in a murine model of leukemia. Sci Transl Med (2021) 13(625):eabg6986. doi: 10.1126/scitranslmed.abg6986 34936380 PMC9116279

[201] Palomba ML. Mei MG. Caimi PF Cortese M Davila M Yadnik G. Abstract CT125: A Phase 1 study to evaluate the safety and tolerability of a combination autologous CD19 CAR T cell therapy (SYNCAR-001) and orthogonal IL-2 (STK-009) in subjects with relapsed or refractory CD19 expressing hematologic Malignancies (NCT05665062). Cancer Res (2023) 83(CT125):CT125. doi: 10.1158/1538-7445.AM2023-CT125

[202] Fraietta JA Lacey SF Orlando EJ Pruteanu-Malinici I Gohil M Lundh S. Determinants of response and resistance to CD19 chimeric antigen receptor (CAR) T cell therapy of chronic lymphocytic leukemia. Nat Med (2018) 24(5):563–71. doi: 10.1038/s41591-018-0010-1 PMC6117613 29713085

[203] Gattinoni L Speiser DE Lichterfeld M Bonini C. T memory stem cells in health and disease. Nat Med (2017) 23(1):18–27. doi: 10.1038/nm.4241 28060797 PMC6354775

[204] Huang J Khong HT Dudley ME El-Gamil M Li YF Rosenberg SA. Survival, persistence, and progressive differentiation of adoptively transferred tumor-reactive T cells associated with tumor regression. J Immunother (2005) 28(3):258–67. doi: 10.1097/01.cji.0000158855.92792.7a PMC2174599 15838383

[205] Krishna S Lowery FJ Copeland AR Bahadiroglu E Mukherjee R Jia L. Stem-like CD8 T cells mediate response of adoptive cell immunotherapy against human cancer. Science (2020) 370(6522):1328–34. doi: 10.1126/science.abb9847 PMC8883579 33303615

[206] Sade-Feldman M Yizhak K Bjorgaard SL Ray JP de Boer CG Jenkins RW. Defining T cell states associated with response to checkpoint immunotherapy in melanoma. Cell (2019) 176(1-2):404. doi: 10.1016/j.cell.2018.12.034 30633907 PMC6647017

[207] Jafarzadeh L Masoumi E Fallah-Mehrjardi K Mirzaei HR Hadjati J. Prolonged persistence of chimeric antigen receptor (CAR) T cell in adoptive cancer immunotherapy: challenges and ways forward. Front Immunol (2020) 11:702. doi: 10.3389/fimmu.2020.00702 32391013 PMC7188834

[208] Sandau MM Kohlmeier JE Woodland DL Jameson SC. IL-15 regulates both quantitative and qualitative features of the memory CD8 T cell pool. J Immunol (2010) 184(1):35–44. doi: 10.4049/jimmunol.0803355 19949092 PMC2957822

[209] Zhang Y Zhuang Q Wang F Zhang C Xu C Gu A. Co-expression IL-15 receptor alpha with IL-15 reduces toxicity *via* limiting IL-15 systemic exposure during CAR-T immunotherapy. J Trans Med (2022) 20(1):432. doi: 10.1186/s12967-022-03626-x PMC9516829 36167591

[210] Gust J Ponce R Liles WC Garden GA Turtle CJ. Cytokines in CAR T cell-associated neurotoxicity. Front Immunol (2020) 11:577027. doi: 10.3389/fimmu.2020.577027 33391257 PMC7772425

[211] Hurton LV Singh H Najjar AM Switzer KC Mi T Maiti S. Tethered IL-15 augments antitumor activity and promotes a stem-cell memory subset in tumor-specific T cells. Proc Natl Acad Sci U S A (2016) 113(48):E7788–E97. doi: 10.1073/pnas.1610544113 PMC5137758 27849617

[212] Jiang Q Li WQ Hofmeister RR Young HA Hodge DR Keller JR. Distinct regions of the interleukin-7 receptor regulate different Bcl2 family members. Mol Cell Biol (2004) 24(14):6501–13. doi: 10.1128/MCB.24.14.6501-6513.2004 PMC434255 15226449

[213] Markley JC Sadelain M. IL-7 and IL-21 are superior to IL-2 and IL-15 in promoting human T cell-mediated rejection of systemic lymphoma in immunodeficient mice. Blood (2010) 115(17):3508–19. doi: 10.1182/blood-2009-09-241398 PMC2867264 20190192

[214] Luo H Su J Sun R Sun Y Wang Y Dong Y. Coexpression of IL7 and CCL21 increases efficacy of CAR-T cells in solid tumors without requiring preconditioned lymphodepletion. Clin Cancer Res (2020) 26(20):5494–505. doi: 10.1158/1078-0432.CCR-20-0777 32816947

[215] Park JH Yu Q Erman B Appelbaum JS Montoya-Durango D Grimes HL. Suppression of IL7Ralpha transcription by IL-7 and other prosurvival cytokines: a novel mechanism for maximizing IL-7-dependent T cell survival. Immunity (2004) 21(2):289–302. doi: 10.1016/j.immuni.2004.07.016 15308108

[216] Vera JF Hoyos V Savoldo B Quintarelli C Giordano Attianese GM Leen AM. Genetic manipulation of tumor-specific cytotoxic T lymphocytes to restore responsiveness to IL-7. Mol Ther (2009) 17(5):880–8. doi: 10.1038/mt.2009.34 PMC2835146 19259067

[217] Shum T Omer B Tashiro H Kruse RL Wagner DL Parikh K. Constitutive signaling from an engineered IL7 receptor promotes durable tumor elimination by tumor-redirected T cells. Cancer Discovery (2017) 7(11):1238–47. doi: 10.1158/2159-8290.CD-17-0538 PMC5669830 28830878

[218] Zeng R Spolski R Finkelstein SE Oh S Kovanen PE Hinrichs CS. Synergy of IL-21 and IL-15 in regulating CD8+ T cell expansion and function. J Exp Med (2005) 201(1):139–48. doi: 10.1084/jem.20041057 PMC2212766 15630141

[219] Kuchen S Robbins R Sims GP Sheng C Phillips TM Lipsky PE. Essential role of IL-21 in B cell activation, expansion, and plasma cell generation during CD4+ T cell-B cell collaboration. J Immunol (2007) 179(9):5886–96. doi: 10.4049/jimmunol.179.9.5886 17947662

[220] Batra SA Rathi P Guo L Courtney AN Fleurence J Balzeau J. Glypican-3-specific CAR T cells coexpressing IL15 and IL21 have superior expansion and antitumor activity against hepatocellular carcinoma. Cancer Immunol Res (2020) 8(3):309–20. doi: 10.1158/2326-6066.CIR-19-0293 PMC10765595 31953246

[221] Stach M Ptackova P Mucha M Musil J Klener P Otahal P. Inducible secretion of IL-21 augments anti-tumor activity of piggyBac-manufactured chimeric antigen receptor T cells. Cytotherapy (2020) 22(12):744–54. doi: 10.1016/j.jcyt.2020.08.005 32950390

[222] Wang Y Jiang H Luo H Sun Y Shi B Sun R. An IL-4/21 inverted cytokine receptor improving CAR-T cell potency in immunosuppressive solid-tumor microenvironment. Front Immunol (2019) 10:1691. doi: 10.3389/fimmu.2019.01691 31379876 PMC6658891

[223] Kagoya Y Tanaka S Guo T Anczurowski M Wang CH Saso K. A novel chimeric antigen receptor containing a JAK-STAT signaling domain mediates superior antitumor effects. Nat Med (2018) 24(3):352–9. doi: 10.1038/nm.4478 PMC5839992 29400710

[224] Cui W Liu Y Weinstein JS Craft J Kaech SM. An interleukin-21-interleukin-10-STAT3 pathway is critical for functional maturation of memory CD8+ T cells. Immunity (2011) 35(5):792–805. doi: 10.1016/j.immuni.2011.09.017 22118527 PMC3431922

[225] Siegel AM Heimall J Freeman AF Hsu AP Brittain E Brenchley JM. A critical role for STAT3 transcription factor signaling in the development and maintenance of human T cell memory. Immunity (2011) 35(5):806–18. doi: 10.1016/j.immuni.2011.09.016 PMC3228524 22118528

[226] Suarez ER Chang de K Sun J Sui J Freeman GJ Signoretti S. Chimeric antigen receptor T cells secreting anti-PD-L1 antibodies more effectively regress renal cell carcinoma in a humanized mouse model. Oncotarget (2016) 7(23):34341–55. doi: 10.18632/oncotarget.9114 PMC5085160 27145284

[227] Harrasser M Gohil SH Lau H Della Peruta M Muczynski V Patel D. Inducible localized delivery of an anti-PD-1 scFv enhances anti-tumor activity of ROR1 CAR-T cells in TNBC. Breast Cancer Res (2022) 24(1):39. doi: 10.1186/s13058-022-01531-1 35659040 PMC9166313

[228] Pan Z Di S Shi B Jiang H Shi Z Liu Y. Increased antitumor activities of glypican-3-specific chimeric antigen receptor-modified T cells by coexpression of a soluble PD1-CH3 fusion protein. Cancer Immunol Immunother (2018) 67(10):1621–34. doi: 10.1007/s00262-018-2221-1 PMC11028056 30078052

[229] Roberts ZJ Vezan R Avanzi MP Locke FL. Abstract CT055: Phase 1/2 primary analysis of ZUMA-6: Axicabtagene ciloleucel (Axi-Cel) in combination With atezolizumab (Atezo) for the treatment of patients (Pts) with refractory diffuse large B cell lymphoma (DLBCL). Cancer Res (2020) 80. doi: 10.18632/oncotarget.9114

[230] Hirayama AV Fiorenza S Gauthier J Voutsinas JM Wu QV Kimble EL. Timing of PD-L1 blockade with durvalumab may affect outcomes of CD19 CAR-T cell therapy for relapsed/Refractory large B-Cell lymphoma. Blood (2022) 140:7447–9. doi: 10.1182/blood-2022-168185

[231] Condomines M Arnason J Benjamin R Gunset G Plotkin J Sadelain M. Tumor-targeted human T cells expressing CD28-based chimeric antigen receptors circumvent CTLA-4 inhibition. PloS One (2015) 10(6):e0130518. doi: 10.1371/journal.pone.0130518 26110267 PMC4482147

[232] Kenderian MD 1 2 SS Ruella MD 1 M Shestova PhD 1 O PharmD 1 MK Kim MD 1 M Porter MD 3 DL. Identification of PD1 and TIM3 as checkpoints that limit chimeric antigen receptor T cell efficacy in leukemia. Biol Blood Marrow Transplantation (2016) 22(3):S19–21. doi: 10.1158/1538-7445.AM2020-CT055

[233] Albelda S. Abstract 3749: Genetic blockade of the protein tyrosine phosphatase SHP1 augments CAR T cell activity against PDL1 expressing solid tumors. Cancer Res (2017) 77::3749. doi: 10.1158/1538-7445.AM2017-3749

[234] Wiede F Lu KH Du X Liang S Hochheiser K Dodd GT. PTPN2 phosphatase deletion in T cells promotes anti-tumour immunity and CAR T-cell efficacy in solid tumours. EMBO J (2020) 39(2):e103637. doi: 10.1371/journal.pone.0130518 31803974 PMC6960448

[235] Kumar J Kumar R Kumar Singh A Tsakem EL Kathania M Riese MJ. Deletion of Cbl-b inhibits CD8(+) T-cell exhaustion and promotes CAR T-cell function. J Immunother Cancer (2021) 9(1). doi: 10.1136/jitc-2020-001688 PMC7813298 33462140

[236] Sutra Del Galy A Menegatti S Fuentealba J Lucibello F Perrin L Helft J. *In vivo* genome-wide CRISPR screens identify SOCS1 as intrinsic checkpoint of CD4(+) T(H)1 cell response. Sci Immunol (2021) 6(66):eabe8219. doi: 10.1101/2021.04.12.439455 34860579

[237] Lv J Qin L Zhao R Wu D Wu Z Zheng D. Disruption of CISH promotes the antitumor activity of human T cells and decreases PD-1 expression levels. Mol Ther Oncolytics (2023) 28:46–58. doi: 10.1016/j.omto.2022.12.003 36654786 PMC9827364

[238] Wiede F Lu KH Du X Zeissig MN Xu R Goh PK. PTP1B is an intracellular checkpoint that limits T-cell and CAR T-cell antitumor immunity. Cancer Discovery (2022) 12(3):752–73. doi: 10.1158/2159-8290.CD-21-0694 PMC8904293 34794959

[239] Si J Shi X Sun S Zou B Li Y An D. Hematopoietic progenitor kinase1 (HPK1) mediates T cell dysfunction and is a druggable target for T cell-based immunotherapies. Cancer Cell (2020) 38(4):551–66.e11. doi: 10.1016/j.ccell.2020.08.001 32860752

[240] Carnevale J Shifrut E Kale N Nyberg WA Blaeschke F Chen YY. RASA2 ablation in T cells boosts antigen sensitivity and long-term function. Nature (2022) 609(7925):174–82. doi: 10.1038/s41586-022-05126-w PMC9433322 36002574

[241] Mai D Johnson O Reff J Fan TJ Scholler J Sheppard NC. Combined disruption of T cell inflammatory regulators Regnase-1 and Roquin-1 enhances antitumor activity of engineered human T cells. Proc Natl Acad Sci U S A (2023) 120(12):e2218632120. doi: 10.1073/pnas.2218632120 36920923 PMC10041166

[242] Lee JW Komar CA Bengsch F Graham K Beatty GL. Genetically engineered mouse models of pancreatic cancer: the KPC model (LSL-kras(G12D/+) ;LSL-trp53(R172H/+) ;Pdx-1-cre), its variants, and their application in immuno-oncology drug discovery. Curr Protoc Pharmacol (2016) 73:14 39 1–14 39 20. doi: 10.1002/cpph.2 PMC4915217 27248578

[243] Liu Y Wu W Cai C Zhang H Shen H Han Y. Patient-derived xenograft models in cancer therapy: technologies and applications. Signal Transduct Target Ther (2023) 8(1):160. doi: 10.1038/s41392-023-01419-2 37045827 PMC10097874

[244] Alicke B Totpal K Schartner JM Berkley AM Lehar SM Capietto AH. Immunization associated with primary tumor growth leads to rejection of commonly used syngeneic tumors upon tumor rechallenge. J Immunother Cancer (2020) 8(2). doi: 10.1136/jitc-2020-000532 PMC7368499 32675310

[245] Duncan BB Dunbar CE Ishii K. Applying a clinical lens to animal models of CAR-T cell therapies. Mol Ther Methods Clin Dev (2022) 27:17–31. doi: 10.1016/j.omtm.2022.08.008 36156878 PMC9478925

